# Renewable synthesis of MoO_3_ nanosheets via low temperature phase transition for supercapacitor application

**DOI:** 10.1038/s41598-024-69765-x

**Published:** 2024-09-03

**Authors:** K. N. Amba Sankar, Lokesh Kesavan, Bikash Saha, M. K. Jyolsnaraj, S. Mohan, P. Nandakumar, Kallol Mohanta, Carita Kvarnström

**Affiliations:** 1 Department of Electronics, PSG College of Arts and Science, Coimbatore, Tamil Nadu 641014 India; 2 Department of Chemistry, Materials Chemistry, University of Turku, Henrikinkatu 2, 20014 Turku, Finland; 3 Solid State Physics Division, Bhabha Atomic Research Centre, Mumbai, 400085 India; 4 Homi Bhabha National Institute, Anushakti Nagar, Mumbai, 400 094 India; 5 Nanotech Research Innovation and Incubation Centre (NRIIC), PSG Institute of Advanced Studies, Avinashi Road, Coimbatore, Tamil Nadu 641004 India; 6 Physical and Materials Chemistry Division, CSIR-National Chemical Laboratory, Pune, 411008 India; 7 Senior Research Scientist, Prophecy Sensorlytics LLC, GN4, Sector V, Salt Lake, Kolkata, West Bengal 700156 India; 8 Department of Physics and Astronomy, University of Missouri, 223 Physics Building, Columbia, MO 65211 USA

**Keywords:** Renewable green synthesis, Very low temperature phase transition, *h-*MoO_3_, *α*-MoO_3_, Mixed phases (*h* and* α*) of MoO_3_, MoO_3_ 2D nanosheets, Supercapacitors, Chemistry, Materials chemistry

## Abstract

2D transition metal oxides have created revolution in the field of supercapacitors due to their fabulous electrochemical performance and stability. Molybdenum trioxides (MoO_3_) are one of the most prominent solid-state materials employed in energy storage applications. In this present work, we report a non-laborious physical vapor deposition (PV**D**) and ultrasonic extraction (US**E**) followed by vacuum assisted solvothermal treatment (V**ST**) route (***DEST***), to produce 2D MoO_3_ nanosheets, without any complex equipment requirements. Phase transition in MoO_3_ is often achieved at very high temperatures by other reported works. But our well-thought-out, robust approach led to a phase transition from one phase to another phase, for e.g., hexagonal (*h-*MoO_3_) to orthorhombic (*α*-MoO_3_) structure at very low temperature (90 °C), using a green solvent (H_2_O) and renewable energy. This was achieved by implementing the concept of oxygen vacancy defects and solvolysis. The synthesized 2D nanomaterials were investigated for electrochemical performance as supercapacitor electrode materials. The *α*-MoO_3_ electrode material has shown supreme capacitance (256 Fg^−1^) than its counterpart *h*-MoO_3_ and mixed phases (*h* and* α*) of MoO_3_ (< 50 Fg^−1^). Thus, this work opens up a new possibility to synthesize electrocapacitive 2D MoO_3_ nanosheets in an eco-friendly and energy efficient way; hence can contribute in renewable circular economy.

## Introduction

Nanometal oxide compounds have attracted considerable interest for the creation of advanced supercapacitors due to their capability to deliver pseudo-capacitance. Two dimensional (2D) nanostructures of transition metal oxides exhibit excellent performance in energy storage applications as they possess high fraction of surface exposed atoms. They have enhanced specific capacitance with better energy density compared to carbon nanomaterials and good chemical stability than conductive polymers^1^. To utilize the potential of metal oxides in this context, it is crucial to regulate their electronic structure, electrical conductivity, and active sites. Introducing oxygen vacancies into metal oxides presents promising opportunities for enhancing these attributes. Therefore, a comprehensive overview of the latest advancements in this field is critically important^2^.

Molybdenum compounds have so far have had a big impact on the world of 2D materials. Similar to graphite, it is layered and may be exfoliated or reconstructed into thin layers. Molybdenum trioxide (MoO_3_) is one of the transition metal oxide material with unique physical and chemical characteristics such as phase transfer and charge transfer ability, cation accommodation efficiency, semi-conductance etc.^3^, thus paving a way to various technological applications in recent times, such as, catalysts, gas sensors, supercapacitors, and batteries. MoO_3_ has fascinating wide band gap and three existing polymorphs phases. These are low temperature metastable monoclinic (*β*-MoO_3_), metastable hexagonal (*h-*MoO_3_) and thermodynamically stable orthorhombic (*α*-MoO_3_) structures^4–7^.

Physical vapor deposition (PVD) is one of the important techniques to deposit MoO_3_ on a chosen substrate because it produces highly crystalline and stratified structures with high yield^3^. In an oxygen-deficient environment, the reduction of MoO_3_ gives rise to several phases of MoO_3-x_ (where 0 < x < 1) with a ReO_3_-type structure, such as Mo_9_O_26_, Mo_8_O_23_, and Mo_4_O_11_. These phases are categorized as part of the Magneli series, characterized by the composition Mo_n_O_3n-1_^8^. The phase transition in MoO_3_ can be achieved through thermal treatments at above 350 °C^9^. *h-*MoO_3_ can be obtained at 440 °C^10^, whereas *β*-MoO_3_ and stable *α*-MoO_3_ phases can be obtained at 600 °C, 750 °C respectively^4^. Among these different phases, *α*-MoO_3_ is getting more attention due to excellent catalytic activity and high electrochemical performance as reported in the literature^11^.

Regulating the physicochemical properties through engineering of oxygen defects is a powerful approach to significantly alter materials' characteristics in a controlled manner. Generally, oxygen defects play a crucial role in the optical and electrical properties of transition metal oxide materials. In the case of oxygen-deficient MoO_3-x_, the presence of oxygen vacancies leads to the formation of energy states within the forbidden optical band gap of the growing thin film, facilitated by the presence of excess molybdenum (Mo) atoms^12–14^. The presence of oxygen vacancies in interlayer spacing followed by phase transition, which in turn will reflect in superior specific surface area, electroactive sites, electrical conductivity. The intercalation offers two times higher pseudocapacitive behavior for MoO_3-x_ than MoO_3_^15^. Hence, oxygen defect mediated phase transition is essential to study in developing desired application. However, these phase transitions are often carried out at high temperatures, thus consuming lot of thermal energy and energy costs. Hence, in overall, it is important to study cost effective, eco-friendly phase transitions to support renewable, recyclable processes.

Based on the prior literature knowledge on MoO_3_ syntheses and characterizations, we decided to develop a facile method (***DEST***) in which we aimed to convert the commercially acquired bulk MoO_3_ material into an amorphous (*am*-) MoO_3-x_ nanomaterial first, by physical vapor deposition (PV**D**), then ultrasonically extract (US**E**) this nanomaterial into suitable green solvents, which will be the resource feed for the phase transition of MoO_3-x_ that we tried to achieve at very low temperature under vacuum assisted solvothermal treatment (V**ST**) conditions. In this work, we report an oxygen defect mediated phase transition (*am* → *h* → *α* MoO_3_) happening just at 60-90 °C, in water or *iso*-propanol, for the first time ever. VST can be done in rotary evaporation mode, which is a very basic technique to extract or remove solvents^16^. This simple, yet fruitive method is proposed for the potential use in the synthesis of metastable *h*-MoO_3_, stable *α*-MoO_3_ and mixed *h* and *α*-MoO_3_ from pre-synthesized *am*-MoO_3-x_ solutions. Moreover, these crystalline phases of MoO_3_ can be finetuned using an appropriate selection of solvents, and operating modalities like temperature, vacuum level, rotation speed etc., during VST. We also believe that our *DEST* synthesis approach could bring down enormous energy and waste solvent management costs while scaling up the production of 2D MoO_3_ nanosheets. Further, we have characterized the *DEST*-synthesized materials to understand the effect of oxygen vacancies and the mechanism of phase transition. The postulates have been detailed using hypothetical premises. Finally, the as-synthesized crystalline phases of MoO_3_ were subjected to a model application; supercapacitor performance, which is an auxiliary study in this work.

Our primary focus in the present work is to convey the larger message to the scientific community that the phase transition of MoO_3_ polymorphs can be done at lower temperatures using green solvents without any sophisticated equipment requirements. For e.g., Jadkar et al*.* reported a method which employed CVD technique with the operation temperature of 1500 ± 50 °C^17^. In their method, ‘Mo’ filament was directly oxidized to *α*-MoO_3_. Also, there was no scope of intermediate phase transitions like, from hexagonal phase (*h-*) and orthorhombic phase (*α*-). Han et al*.*’s method required an electrospinning equipment and again high temperature, up to 300 °C was needed to produce *α*-MoO_3_ from its precursor^18^. Whereas, our work involves in converting amorphous MoO_3-x_ into* h*-MoO_3_, *α*-MoO_3_ and mixed phases (*h *and* α*) of MoO_3_ at the maximum 60–90 °C using either water or *iso*-propanol in a simple vacuum distillation unit. The new insights we are aiming to provide here is the theoretical background of oxygen vacancies and its refilling by solvolysis, which can yield different crystalline phases of MoO_3_ practically.

The other important reason why we study the phase transitions in MoO_3_ crystal systems is that different phases of MoO_3_ crystals could possess varying physico-chemical properties. Materials researchers would always want to exploit these properties, to suit their application that they are developing, for e.g., catalysis, adsorbents, composites, charge storage etc. Hence, we chose to carry out capacitance measurement studies in this present work. Making comparisons are part of research exercise. Thus, we compared the MoO_3_ polymorphs for their electrochemical performance. As these polymorphs were prepared by a new route, we were inquisitive of knowing their electrochemical properties. We believe that our *DEST* synthesis approach could bring up new properties in the crystalline structure of 2D MoO_3_ nanosheets. Therefore, it would offer a vibrant supercapacitor material in energy storage applications for the future. Also, our work can contribute to a renewable, circular economy in a longer run.

## Results and discussion

### Structural determination of MoO_3_ polymorphs

*DEST*-synthesized MoO_3_ samples such as (1) *am*-MoO_3-x_ solvolyzed at 60 °C in water (MO_w60_), (2) *am*-MoO_3-x_ solvolyzed at 90 °C in water (MO_w90_) and (3) *am*-MoO_3-x_ solvolyzed at 90 °C in *iso*-propanol (MO_ipa90_) were subjected to detailed functional group analyses (FT-IR, Raman, UV–Vis, XRD, XPS), after their phase transition, to investigate the chemical bonding states between molybdenum and oxygen atoms.

**Figure 1:**
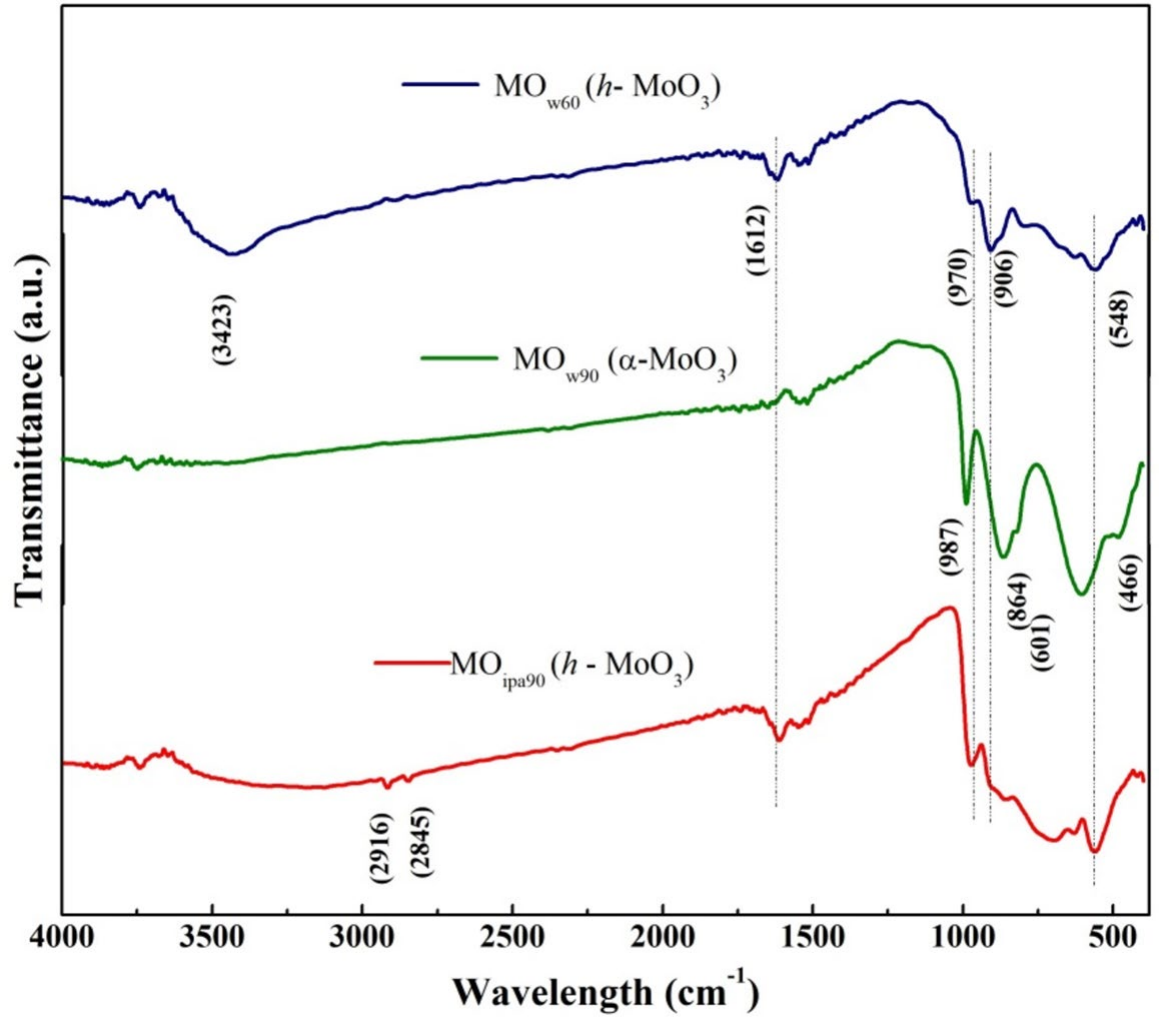
FTIR spectra of *DEST*-made MoO_3_ polymorphs materials.

FT-IR spectra (Fig. 1) confirmed that MO_w60_ and MO_ipa90_ were exhibiting almost similar characteristic responses like (i) a shallow peak at 3423 cm^−1^ corresponding to H–OH stretching vibration of firmly bounded water molecules in the sample^19^, (ii) less intense peaks at 2916 cm^−1^, 2845 cm^−1^ denoting C–H stretching, (iii) a prominent peak at 1612 cm^−1^ attributed to H–O–H bending vibration, (iv) then finger print region with some clear peaks at 970 cm^−1^, 906 cm^−1^ due to stretching of Mo=O double bond vibrations of hexagonal phase^20^, and a peak at 548 cm^−1^ due to single oxygen atom interaction with three molybdenum atoms^21^ (Fig. 1). MO_w60_ and MO_ipa90_ samples having two peaks present at 2916 cm^−1^ and 2845 cm^−1^ were attributed to –CH_2_ vibration^22^ caused by any organic impurities in H_2_O or *iso*-propanol.

On the other hand, MO_w90_ sample did not show any peaks at higher wavenumbers (from 3500 cm^−1^ to 1700 cm^−1^) but at 1612 cm^−1^ and the finger print region, similar to MO_w60_ (Fig. 1). The finger print region in MO_w90_ was slightly blue shifted to (a) 987 cm^−1^, (b) 864 cm^−1^, (c) 601 cm^−1^, and (d) 466 cm^−1^, attributable to (a) Mo=O stretching vibration which is representing the stable, layered orthorhombic MoO_3_ phase, (b) orthorhombic MoO_3_ structure having Mo–O–Mo bonding with stretching vibrations of O_3_ atoms^23^, (c) Mo–O vibrations^21^, (d) Mo_2_–O_4_ bonding due to solvolyzing H_2_O molecules, respectively. From these results, it was confirmed that the samples MO_w60_ and MO_ipa90_ were in hexagonal crystalline structure (*h*-MoO_3_) and MO_w90_ was in orthorhombic crystalline structure (*α*-MoO_3_). In order to confirm the phase transition further, the Raman Spectroscopy was applied next.

**Figure 2:**
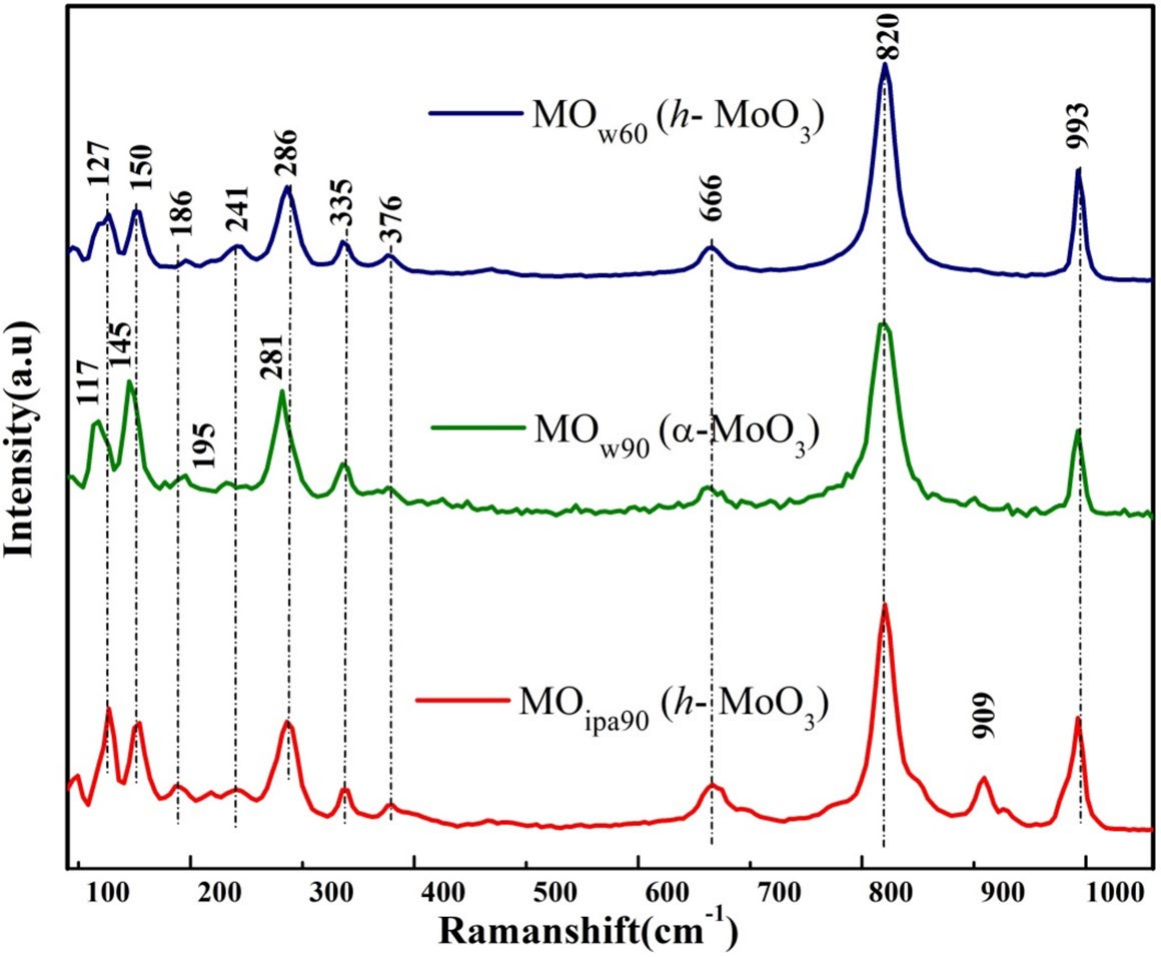
Raman spectra of *DEST*-made MoO_3_ polymorphs materials.

The micro-Raman spectra of all three MoO_3_ samples (MO_w60_, MO_w90_, MO_ipa90_) were collected under 523 nm excitation and recorded in the range from 1100 to 100 cm^−1^ as shown in Fig. 2. The bands appeared between 100 to 400 cm^−1^ and 600 to 1000 cm^−1^ were representing bending, stretching vibrations of Mo–O/Mo=O. These are the lattice modes giving rise to the bands in lower region of MoO_6_ octahedra in *h*-MoO_3_ and *α*-MoO_3_, respectively^24^. The peaks appeared from 100 to 300 cm^−1^ were due to the MoO_6_ skeleton base structure, the peaks between 300 and 700 cm^−1^ were assigned to O–Mo–O bonds and the absorption at high wave number range, 700 to 1000 cm^−1^, corresponded to Mo–O–Mo bonding. A well-defined sharp peak present at 820 cm^−1^, along with other main peaks 666 and 993 cm^−1^ were reflecting the fingerprint region of the stable *α*-MoO_3_ phase^24–26^. A tiny peak at 909 cm^−1^ present in MO_w90_, MO_ipa90_ samples could be an indication of shared identity of a particular crystalline phase between these two samples. The detailed Raman vibrational analysis of bonds for *h*-MoO_3_ and *α*-MoO_3_ in comparison with literature evidences is shown in supplementary information file (SI); Tables S1 and S2. From this, the formation of *h*-MoO_3_ (MO_w60_, MO_ipa90_) and *α*-MoO_3_ (MO_w90_) in our experimental conditions were confirmed. Further to determine the electronic states/band gaps in these polymorphs, the synthesized MoO_3_ samples (MO_w60_, MO_w90_, MO_ipa90_) were subjected to UV–Vis absorption spectroscopy.

**Figure 3:**
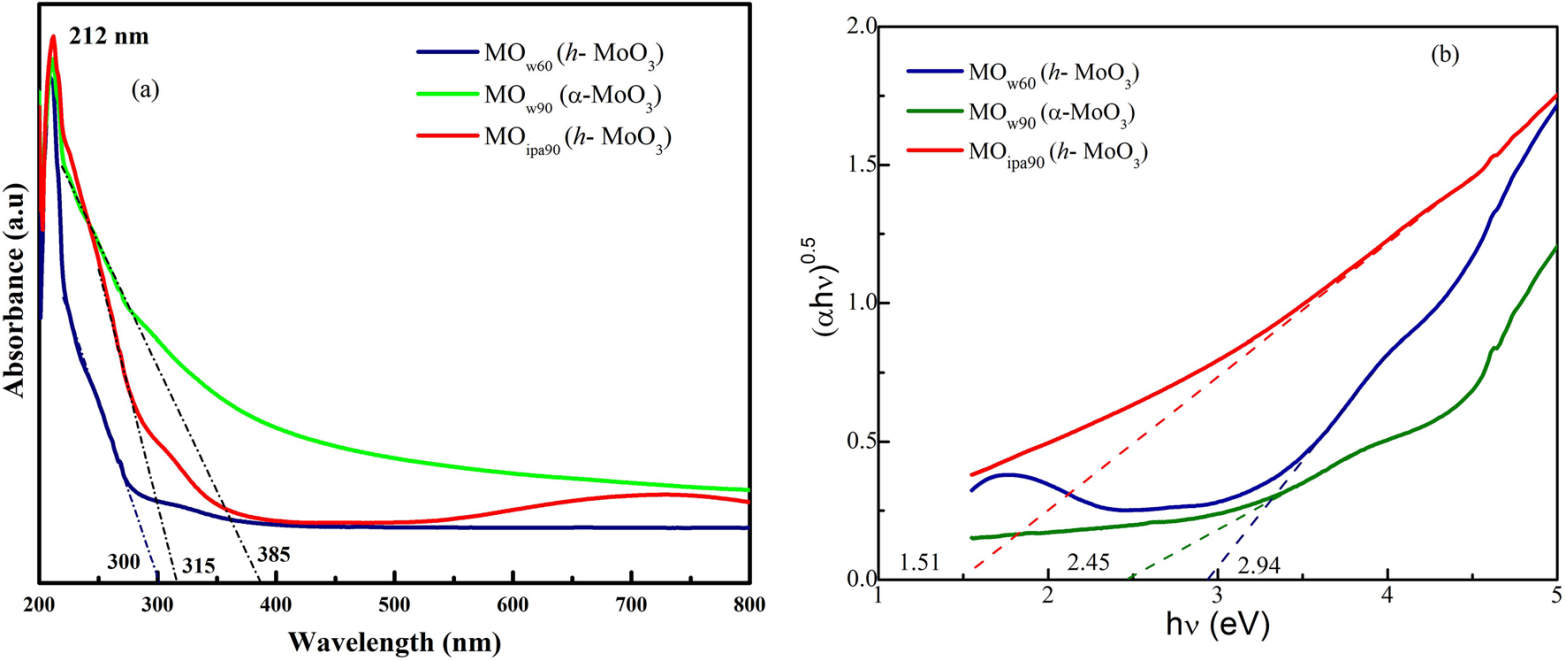
UV–Vis spectra of *DEST*-made MoO_3_ polymorphs materials. (**a**) Absorbance spectra, (**b**) Tauc plot for finding E_g_.

The UV–Vis spectroscopic study, as illustrated in Fig. 3a, provides detailed insights into the optical properties of the samples, MO_w60_, MO_w90_, and MO_ipa90_. A distinct excitonic absorption peak at 212 nm was observed in all three samples, indicating the presence of 2D MoO_3_ nanosheets^27^. This characteristic excitonic absorption is a hallmark feature of 2D materials. Furthermore, additional band edge absorptions were observed at different wavelengths for each sample: 300 nm for MO_w60_ (*h*-MoO_3_), 385 nm for MO_w90_ (*α*-MoO_3_), and 315 nm for MO_ipa90_ (*h*-MoO_3_). These characteristic absorption wavelengths indicated variations in the electronic properties and band structures in these samples. Thus, confirming different phases of MoO_3_. Figure 3b showcases the determination of band gaps (E_g_) through Tauc plot analysis using the UV–Vis spectrum. The calculated E_g_ values for MO_w60_ (*h*-MoO_3_), MO_w90_ (*α*-MoO_3_), and MO_ipa90_ (*h*-MoO_3_) were 2.94 eV, 2.45 eV, and 1.51 eV respectively. These values did match with those values reported in literature by Lei Zheng et al.^10^, Hanmei Hu et al.^28^, and Yuehong Song et al.^29^, providing validation for the accuracy of the experimental measurements.

However, some more clarity was needed to distinguish MO_w60_ from MO_ipa90_ as though they showed similar IR, Raman spectral lines, their UV–Vis spectral data revealed that there were some minute variations between them. Also, in Raman Spectroscopic data, there was an ambiguity in identifying crystalline phases, as all three samples showed the distinct peaks at 666 cm^−1^, 820 cm^−1^ and 993 cm^−1^. To sort this out, we went for an extensive crystal structural analysis of these MoO_3_ polymorphs using XRD technique.

### Crystal structural analysis of MoO_3_ phases

The most important analysis to prove the phase transition in as-synthesized MoO_3_ samples was X-ray diffraction studies (XRD). Figure S1 shows the XRD patterns of commercially purchased stock MoO_3_ (bulk powder), physical vapor deposited MoO_3-x_ (nanopowder), and then recrystallized MoO_3_ samples from water (MO_w60_, MO_w90_) and isopropanol (MO_ipa90_), along with simulated diffraction patterns of *h*-MoO_3_ and *α*-MoO_3_. This direct comparison allowed us to assess the peak positions and intensities of the corresponding phases.

The thin films of MoO_3_ (thickness of 150 nm) deposited on glass substrates by PVD, are usually amorphous and sub-stoichiometric (*am*-MoO_3-x_) in nature. The absence of sharp peaks and presence of broad hump (26.17°) in the XRD pattern confirmed the amorphous (*am*-) nature of as-deposited thin films (Fig. S1). This hump was due to weak diffraction, suggesting that the film possesses a combination of amorphous and crystalline characteristics (64% amorphous and 36% crystalline).

The crystallinity percentage was calculated using the following formula;

$${\text{Crystallinity }}\;(\%) = \frac{{\text{Area of crystalline peaks }}}{{{\text{Area of crystalline peak}} + {\text{Area of amorphous peak}}}} \times 100$$

Contrary to *am*-MoO_3-x_, the recrystallized MoO_3_ samples; MO_w60,_ MO_w90,_ MO_ipa90_, were exhibiting sharp distinct peaks in the XRD pattern, suggesting phase transition and enhanced crystallinity (Fig. S1, 4). The samples recrystallized using water (MO_w60_) exhibited a phase transition from amorphous (*am*-MoO_3-x_) to hexagonal structure (*h*-MoO_3_). However, with an increase in recrystallization temperature (MO_w90_), a complete phase transition from amorphous (*am*-MoO_3-x_) to thermodynamically stable orthorhombhic structure (*α-* MoO_3_) was observed. We also believe that this transition should have happened via the intermediate, which is metastable *h*-MoO_3_ form, as we already know that *h*- structure was the resultant phase at 60 ° C in H_2_O. Conversely, in the case of MoO_3_ sample recrystallized using *iso*-propanol (MO_ipa90_), a mixed phases of *h* and *α*- MoO_3_ product was obtained, suggesting an initial phase transition from *am*-MoO_3 -x_ to *h*-MoO_3_, followed by a partial phase transition from *h*-MoO_3_ to *α*-MoO_3_ occurred in this sample. The measured diffraction patterns of MoO_3_ phases were analyzed by using the Rietveld refinement technique (assisted by FULLPROF computer program).

**Figure 4:**
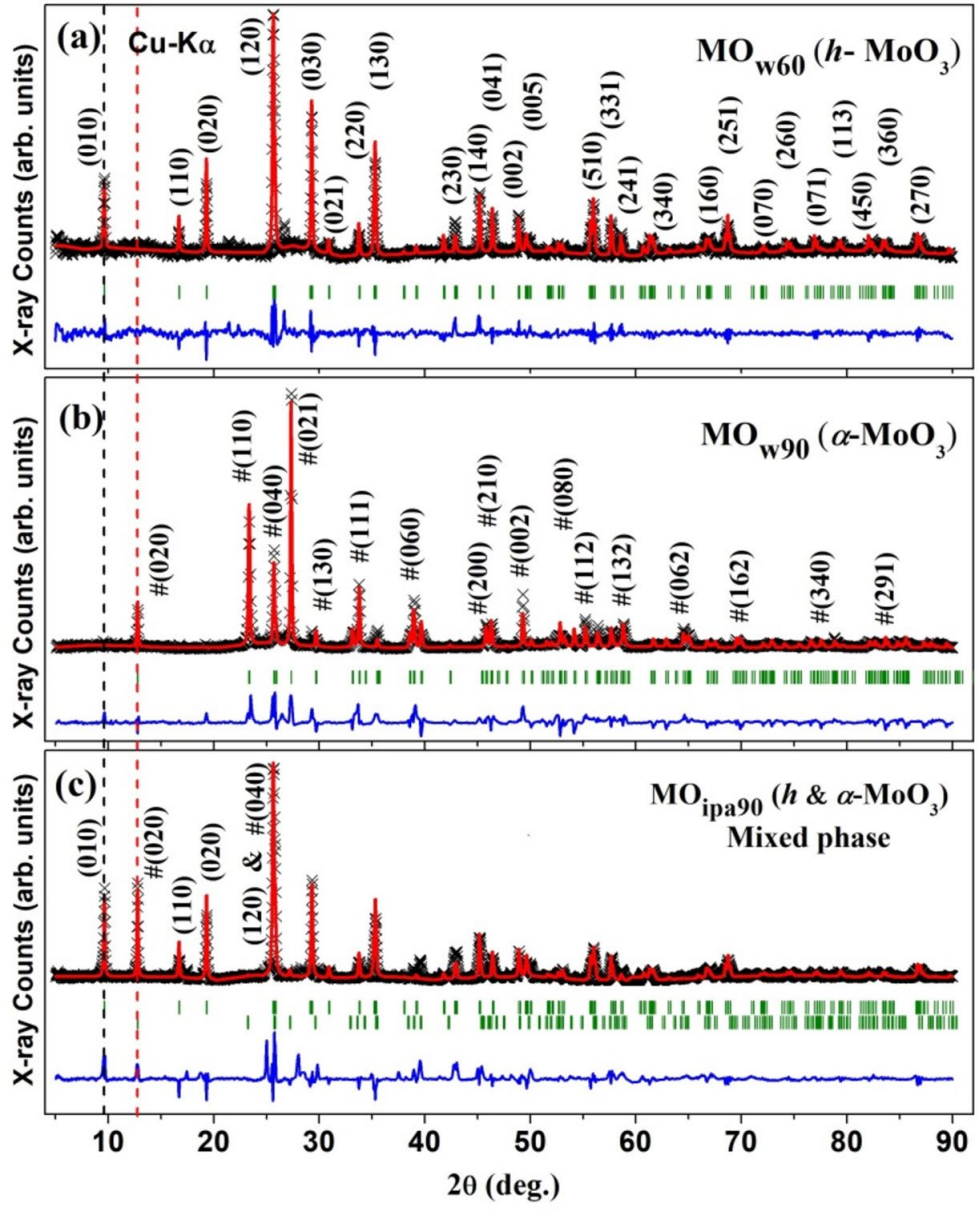
X-ray diffraction patterns refined by Rietveld method for *DEST*-made MoO_3_ polymorphs materials: (**a**) MO_w60_ (*h*-MoO_3_), (**b**) MO_w90_ (*α*-MoO_3_), (**c**) MO_ipa90_ (mixed phases of *h* and *α*-MoO_3_). Black and red lines (through the data points) are representing the experimental and the calculated patterns, respectively. The difference between the experimental and the calculated patterns is shown by the blue lines at the bottom of each panel. The vertical green bars represent the allowed nuclear Bragg positions.

The Rietveld analysis of the XRD of MO_w60_ sample, synthesized at 60 °C in H_2_O, revealed a pure single-phase of *h*-MoO_3_, confirmed by the space group P 63/m and space group number 176 (Fig. 4a). This analysis counted the lattice parameters of *h*-MoO_3_ as follows: *a* = *b* = 10.6133(2), *c* = 3.7243(3), *α* = *β* = 90°, and *γ* = 120°. The fractional coordinates of the atomic positions are presented in Table S3.

**Figure 5:**
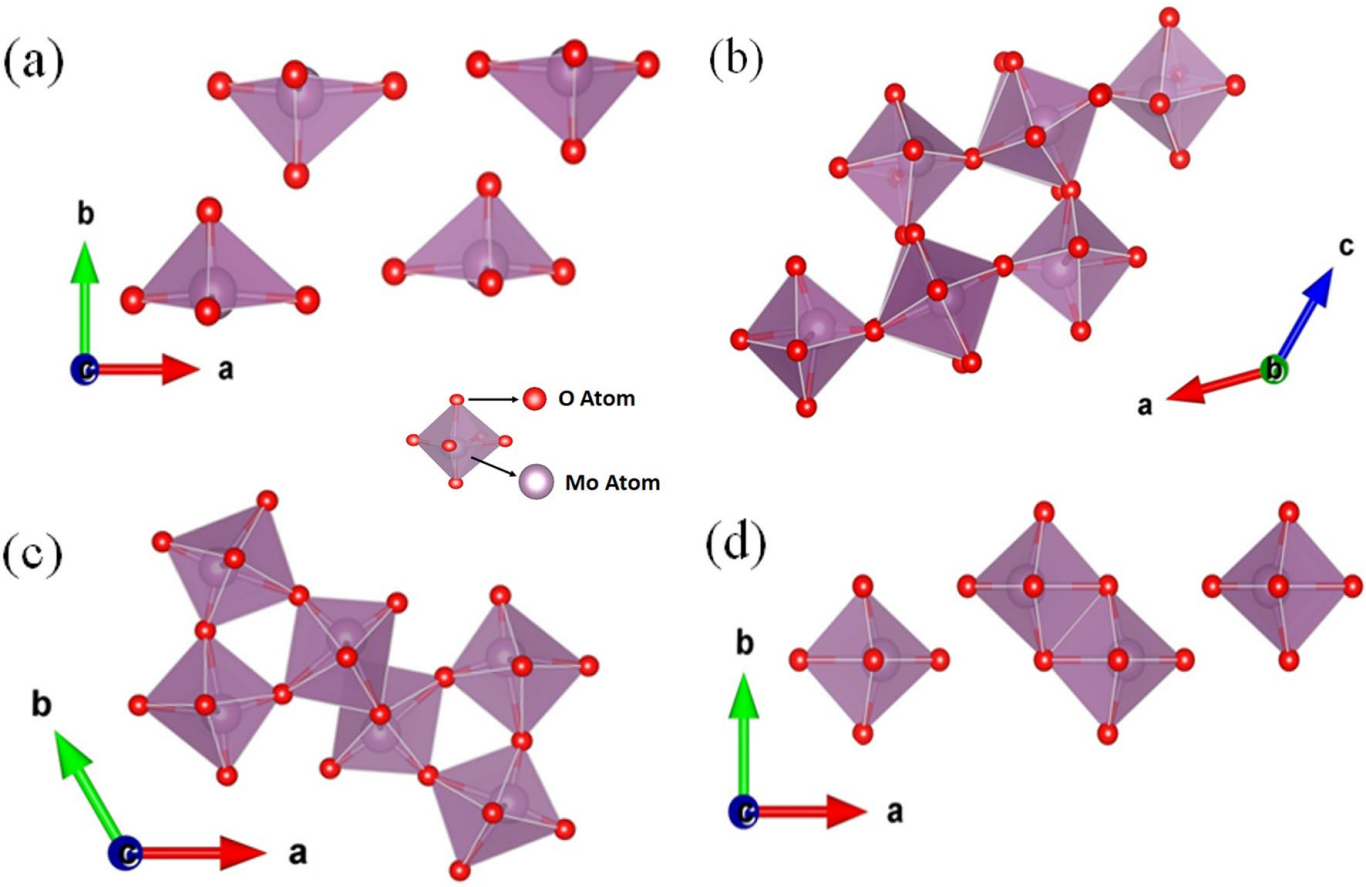
A model unit cell crystal structure of (**a**) MoO_3_, (**b**) *β*-MoO_3_, (**c**) *h*-MoO_3_ and (**d**) *α*-MoO_3_.

This analysis further revealed that the Mo ions reside in octahedral arrangement with the O atoms (Fig. 5a) and no monoclinic *β*-MoO_3_ structure (Fig. 5b) was obtained with any of the synthesized samples (MO_w60_, MO_w90_, MO_ipa90_). The hexagonal crystalline structure (*h*-MoO_3_) of the MO_w60_ sample possess 1D tunnels along crystallographic *c*-axis, which is made up of the zigzag chains of MoO_6_ octahedra, the very building block of the crystal (Fig. 5c).

The Rietveld analysis of the XRD of MO_w90_ sample, synthesized at 90 °C in H_2_O, showed again a single-phase, which is thermodynamically stable orthorhombic crystal symmetry (*α*-MoO_3_), confirmed by the space group P n m a (Fig. 4b). The lattice parameters of *α*-MoO_3_ phase were found to be *a* = 3.9591(2), *b* = 13.8611(6), *c* = 3.7243(3), *α* = *β* = *γ* = 90°. The fractional coordinates of the atomic positions are presented in Table S3. The orthorhombic structure of* α*-MoO_3_ consists of layered planes of the foundational octahedron (*oh*-MoO_6_) units, in double layer form (Fig. 5d). These stacked bilayers of distorted MoO_6_ octahedral units arranged in an ABAB… pattern with edge-sharing zigzag rows and corner-sharing rows, along the [001] and [100] directions.

The Rietveld analysis of the XRD of MO_ipa90_ sample, synthesized at 90 °C in *iso*-propanol ((CH_3_)_2_CHOH) confirmed the presence of (010) and #(020) of *h*-MoO_3_ and *α*-MoO_3_ phases respectively (Fig. 4c), indicating the composition of mixed phases, but predominately composed of* h*-MoO_3_ (space group P 63/m and space group number 176). This analysis (considering both phases) further suggested that the mixed phase contains 72.6% of *h-*MoO_3_ and 27.4% of *α*-MoO_3_. The refined crystal structural parameters of mixed phase are as follows (Table S4): *a* = *b* = 10.6134(2), *c* = 3.7207(3), *α* = *β* = 90°, and *γ* = 120° for *h*-MoO_3_, and *a* = 3. 9832(3), *b* = 13.8673(5), *c* = 3.7192(4), *α* = *β* = *γ* = 90° for *α*-MoO_3_.

Thus, the XRD measurements have confirmed the phase transitions occurred in as-synthesized MoO_3_ samples, very lucidly and proved that they are polymorphs. XRD data also nullified the ambiguity that we had from UV–Vis and Raman spectral data, that (i) whether the phase change happened in MO_w60_ and MO_ipa90_ are same or not, and (ii) the formation of *α*-MoO_3_ in MO_w90_. Henceforth, we mark the polymorph of MO_ipa90_ as ‘*h* and* α*-MoO_3_’ instead of just ‘*h*-MoO_3_’. Therefore, the facile vacuum assisted solvothermal (VST) approach has yielded a very nice phase transition (*am → h → α*) even at very low temperature in the presence of a protic, green solvent like water.

### Surface morphology and topography analysis

**Figure 6:**
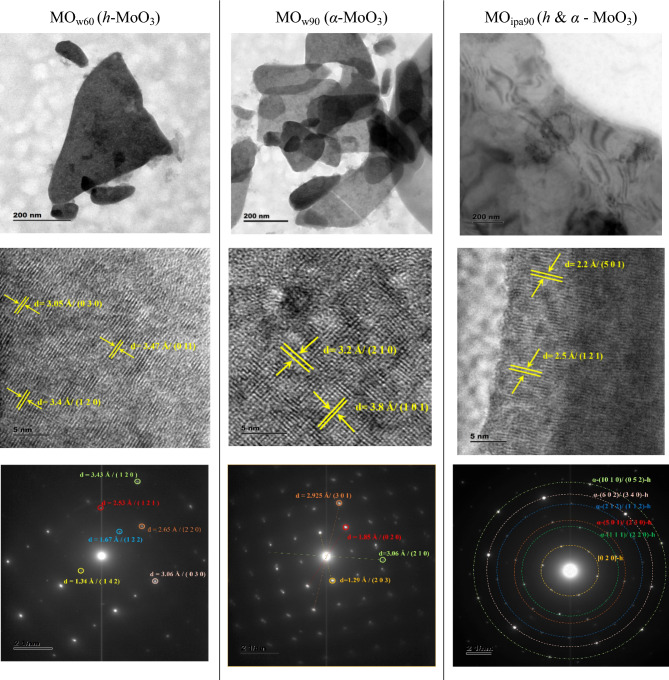
HRTEM images of *DEST*-made MoO_3_ polymorph 2D nanosheet materials.

The *h*-MoO_3_ and *α*-MoO_3_ represent two distinct polymorphs of MoO_3_ with unique structural and electronic properties. Understanding their surface morphology and topography is crucial for exploring their potential applications further. High Resolution Transmission Electron Microscopy (HRTEM) analysis was employed to understand the surface morphology and crystal structure of MoO_3_ polymorphs synthesized by *DEST* method. Images of low magnification, lattice fringes and SAED pattern of the materials (MO_w60_, MO_w90_, MO_ipa90_) are shown in Fig. 6. All the samples were showing a two-dimensional structure in few hundreds of nanometer size. The interplanar spacing analysis of MoO_3_ polymorphs, conducted in lattice fringe images of HRTEM provides crucial insights into the material's structural characteristics at the atomic level. By measuring the distances between adjacent crystal planes along various crystallographic directions, the arrangement of atoms within the MoO_3_ lattice can be understood.

The obtained interplanar spacing results for hexagonal molybdenum trioxide (*h*-MoO_3_) of MO_w60_, were d = 3.47 Å (011), d = 3.4 Å (120), and d = 3.05 Å (030). These (011), (120), and (030) planes confirmed the unique arrangement of atoms within the hexagonal lattice of MoO_3_. The orthorhombic molybdenum trioxide (*α*-MoO_3_) of MO_w90_ exhibited interplanar spacings of d = 3.2 Å (210) and d = 3.8 Å (101). The interplanar spacing of 3.2 Å along the (210) crystallographic plane indicated the short distance between adjacent planes oriented in this direction, revealing the compact nature of the atomic arrangement within this plane. Conversely, the interplanar spacing of 3.8 Å along the (101) plane signified a larger separation between adjacent planes. These findings shed light on the anisotropic nature of *α*-MoO_3_, where the interplanar spacings vary along different crystallographic directions. The mixed-phases of MO_ipa90_ (*h* and* α*-MoO_3_) showed interplanar spacings of d = 2.2 Å (501) and d = 2.5 Å (121), which was smaller than what pure, single phase *h*-MoO_3_ and *α*-MoO_3_ showed.

The bright pointed diffraction spots appeared in Selected Area Electron Diffraction (SAED) pattern confirmed high order of crystallinity in all three samples (MO_w60_, MO_w90_, MO_ipa90_) (Fig. 6). From SAED pattern, the crystalline phases of MO_w60_, MO_ipa90_, were confirmed as hexagonal (*h*-MoO_3_) structure. On the other hand, MO_w90_ was confirmed to be orthorhombic (*α*-MoO_3_) crystal structure. The samples showed different types of lattice fringes with respect to oxygen defects formed during heat treatments (w60, w90, ipa90) under vacuum. The crystal structures of MoO_3_ became irreversibly distorted as oxygen was removed initially from the lattice structure, followed by its refilling and the intercalation of H^+^ ions from solvents^30,31^. This proved our claim again that the *DEST* method could efficiently bring a phase transformation even at very low temperature providing optimized solvothermal conditions (VST) are used.

**Figure 7:**
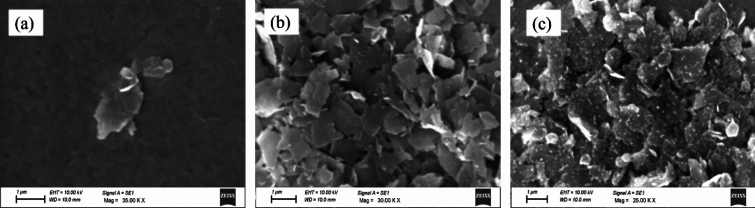
SEM images of *DEST*-made MoO_3_ polymorph 2D nanosheet materials: (**a**) MO_w60_ (*h*-MoO_3_), (**b**) MO_w90_ (*α*-MoO_3_), (**c**) MO_ipa90_ (*h* and *α*-MoO_3_).

**Figure 8:**
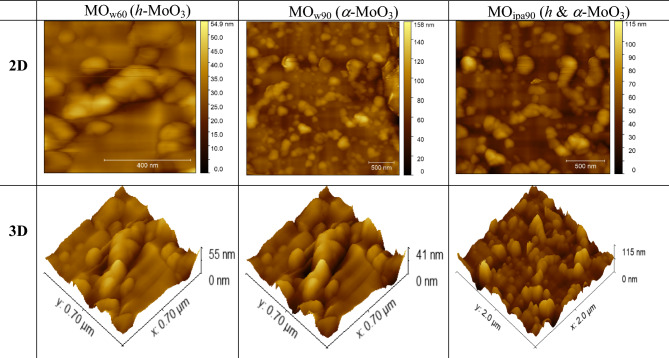
AFM images of *DEST*-made MoO_3_ polymorph 2D nanosheet materials: (**a**) MO_w60_ (*h*-MoO_3_), (**b**) MO_w90_ (*α*-MoO_3_), (**c**) MO _ipa90_ (*h* and *α*-MoO_3_).

Further, we have also investigated the microscopic visuals of 2D nanosheets of *h*-MoO_3_ (MO_w60_), *α*-MoO_3_ (MO_w90_) and mixed phases (*h* and* α*-MoO_3_) of MO_ipa90_, using SEM and AFM techniques as shown in Figs. 7a–c and 8 respectively.

The scanning electron microscopy (SEM) images clearly revealed sheet-like structures with diameters ranging from 1 to 2 microns for all the samples, effectively suggesting two-dimensional (2D) morphology (Fig. 7). Also, the images suggested that the sheets were formed uniformly in dimension, thus our synthesis method offers the feasibility of controlling the dimensions of the MoO_3_ polymorphs at the micron scale level. This observation highlights the successful fabrication of MoO_3_ polymorphs in our desired 2D form which holds significant implications in potential applications. Figure 8 illustrates AFM images of MoO_3_ polymorphs. AFM imaging provided a high-resolution representation of the topography of the samples; MoO_3_ nanoparticles. From the images, we can clearly see the individual nanoparticles appearing as distinct features on the substrate. This allows us to determine the size, shape and arrangement at the nanoscale.

**Figure 9:**
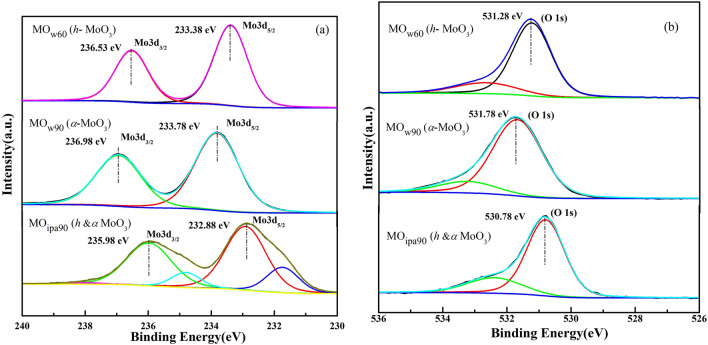
XPS spectra of *DEST*-made MoO_3_ polymorph 2D nanosheet materials (**a**) Mo-3d core level region, (**b**) O-1s region.

Further, the oxidation states of Mo in the material and chemical bonding in the surface of samples (MO_w60_, MO_w90_, MO_ipa90_) were investigated by the X-ray Photoelectron Spectroscopy (XPS) (Fig. 9a, b). The full energy survey spectrum (Fig. S2) of MoO_3_ polymorphs confirmed the presence of Mo^*4p*^, Mo^*3d*^, C^*1s*^, Mo^*3p*^ and O^*1s*^ peaks at their characteristic binding energies that validated the identity of MoO_3_ in the sample.

In Fig. 9a, the deconvolution of the Mo spectrum scan revealed distinct peaks at (236.53 eV, 233.38 eV)^32,33^, and (236.98 eV, 233.78 eV)^19,32,34^, for MO_w60_ and MO_w90_ respectively. These peaks correspond to the spin–orbit doublets (3d_5/2_ and 3d_3/2_) of Mo^6+^, thereby confirming the presence of MoO_3_. However, these peaks slightly shifted to low binding energies (235.98 eV, 232.88 eV) for MO_ipa90_^6,10,35,36^. This shift is due to subtle differences in the local chemical environment which influences the binding energies of Mo-related peaks in the XPS spectra^37,38^. O-1s peaks (Fig. 9b) also showed similar shift (531.28 eV, 531.78 eV, 530.78 eV for MO_w60_, MO_w90_, MO_ipa90_), indicating variations in the oxygen bonding environment among different polymorphs.

To be specific, Table S5 shows that the Mo-3d_3/2_, Mo-3d_5/2_, O-1s peak positions, shifted 0.45 eV. 0.40 eV, 0.5 eV respectively, between the metastable *h*- and most stable *α*- phases of MoO_3_. Similarly shifts of 0.55 eV, 0.50 eV, 0.50 eV noticed for the same peaks correspondingly, between the metastable *h*- and mixed phases (*h* and *α*-MoO_3_) of the material. Further clear shifts of 1 eV, 0.9 eV, 1 eV were observed for the above-mentioned peaks respectively, between the most stable *α*-phase and mixed phases (*h* and* α*-MoO_3_) of the material. Overall, the changes in surface chemistry alter the electron density near the surface, influencing the binding energies of photoelectrons, which resulted in XPS peak shifts.

We believe that *iso*-propanol mediated vacuum assisted solvothermal step (VST) could have caused different oxygen defect in MoO_3_ than the water mediated synthesis. Oxygen defect creates low valent Mo ions, if these low valent ions haven’t regenerated into Mo^6+^ completely, that could cause a slight shift in the binding energy. We also knew that the MO_*ipa90*_ produced mixed phases of *h-*MoO_3_ and* α*-MoO_3_, thus *iso*-propanol acts very differently from water which produced solely the* α*-MoO_3_ at 90 °C.

### Hypothetical insights into the mechanism of phase transition in MoO_3-x_ (*am* → *h* → *α*-MoO_3_) under VST conditions

Our aim was to achieve the phase transition at very low temperature using physico-chemical method rather than widely reported high temperature-physical methods. In physical methods, MoO_3_ is annealed at very high temperature (300–1500 °C) in flow furnaces or chambers, for its phase transition. Thus, consumes lot of energy during its production. We wanted to bring down this energy cost, by introducing a vacuum assisted solvothermal treatment (VST), where careful application of solvent can do a phase transition wonder. This very unique approach and the results, motivated us to discern the mechanistic details based on hypothetical premises.

In our studies, defects implantation has been adopted as a nuanced technique for modifying the crystalline phase and its physico-chemical properties, in order to achieve desired energy storage application from it. Defects created in various locations of the lattices, could adjust band structure, electron density and bonding at different degrees. Oxygen vacancies, a kind of intrinsic defect in metal oxides, is one of the most preferred fine tuning at crystal level, in materials designing. Through controlled removal of oxygen atoms from the lattices of metal oxides, the lattices become disordered and low valence metal ions are created. Further, these low-valent metal ions will look for foreign ions or oxygen atoms present in the solution/environment to make new bonding to stabilize themselves. So that, the free energy of the crystal system can be brought down, for better stability. Thus, paving a way for phase transition in the crystalline materials. This will have a major impact on the crystal structure and its inherent characteristics^39^.

Oxide materials are highly prone to induced oxygen vacancy defects, especially when the metal ions are at higher oxidation state. In our case (MoO_3_), the Mo is at +6 oxidation state, that can be reduced to lower oxidation states, e.g., +4, by creating oxygen vacancies in thermal or chemical environment. It is well-known fact that MoO_3_ could lose oxygen if it is heated under vacuum or other reducing environment conditions. Under these conditions, it should be noted that MoO_3_ can quickly break down into lesser oxides^40–42^, where the stoichiometry deviates from the ideal MoO_3_ composition into MoO_3-x_, due to the absence of oxygen atoms. These lesser oxides are known to have empirical compositions between MoO_3_ and MoO_2_, leading to the formation of low valent Mo^4+^ ions. Further, these low valent Mo^4+^ ions can be stabilized by supplying protons and the oxygen vacancies can be refilled by some fractions of oxygen from the protic solvents (Brønsted acids), to get back MoO_3_. This was the theoretical basis of our experiment and we supplied protons (H^+^) as foreign ions from water and *iso*-propanol, to quench the Mo^4+^ ions, for the regeneration MoO_3_ with modified phase.

In this present work, we believe that the vacuum environment during PVD step, could have created some oxygen vacancies (Vo^i^) already, with the formation of sub-stoichiometric molybdenum oxide, MoO_3-x_ (150 nm thickness), but without crystallinity (Fig. S1). Hence, to complete the recrystallization, we have taken the VST approach, after the MoO_3-x_ extraction step (USE). During VST also, there might be a creation of oxygen vacancies (Vo^ii^) due to vacuum, with the formation of MoO_3-(x+y)_. If the thickness of the MoO_3-x_ thin film deposition increased up to 350 nm, then positively charged structural defects would also increase due to the concentration of oxygen vacancies^43–45^.

We denote these oxygen vacancies as,

1. Vo^i^—Primary oxygen vacancies: Intermediate—amorphous (*am*-) MoO_3-x_, x—fraction of oxygen defected created during PVD.
2. Vo^ii^—Secondary oxygen vacancies: Intermediate—MoO_3-(x+y)_; y—fraction of oxygen defected during VST.

Later, these oxygen defective intermediates were oxygen refilled to some extent and stabilized by protons through thermo-solvolysis at VST step. In VST, we have chosen the primary protic solvent, H_2_O, as our reagent, which is green (eco-friendly, low cost, renewable) in nature. In order to evaluate its comparative performance, we have also employed *iso*-propanol ((CH_3_)_2_CHOH), another weak protic solvent for solvolysis. In general, water is a highly polar solvent, which makes it effective for dissolving ionic compounds and facilitating reactions involving charged species. Many oxidation reactions are thermodynamically favorable in aqueous environments due to the high dielectric constant and hydrogen bonding capabilities of water. These properties stabilize charged intermediates and transition states, lowering the activation energy barrier for the reaction. After the oxidation reaction, water can be easily separated from the reaction mixture by distillation, evaporation, or other separation techniques. This simplifies product purification and reduces processing costs. *iso*-propanol is also a moderately polar solvent which can dissolve both polar and non-polar substances and again can stabilize the intermediates through hydrogen bonding or solvation effects, thereby influencing the reaction pathway and product distribution. Here, we mostly rely on the Brønsted–Lowry acidity and dissociation constant of the solvents to impact the hydrolysis of oxides.

The vacuum assisted heating (VST) creates the secondary oxygen defects followed by proton-hungry molybdate anions (MoO_4_^2−^) in the presence of these low-boiling solvents. What these solvents basically do is that, they push/shift the reaction equilibrium towards the right side of the equation, thereby inducing the formation of these anion intermediates according to Le Chatelier’s principle of chemical equilibrium. Thus, we end up with regeneration of MoO_3_ from the decomposition of molybdic acid, but with modified phase. Based on the product materials’ characterization results, it was confirmed that water mediated VST did yield a successful phase transition (*am → h* → *α*) in MoO_3-x_, whereas *iso*-propanol mediated VST delivered a partial phase transition (*am → h* → *h* and* α*), leaving a mixed phase product material. However, both the solvents yielded phase transition from *am → h* in MoO_3-x_ thoroughly and effectively.

We reasoned out that the physico-chemical properties of solvents play a major role in facilitating phase transition in MoO_3-x_ during VST. For e.g., Water is a more protic solvent than *iso*-propanol, because it has ten thousand times higher dissociation constant (H_2_O: K_a_ = 1 × 10^−14^) than *iso*-propanol ((CH_3_)_2_CHOH): (K_a_ = 1 × 10^−18^). Hence, water would release more protons in a short span of time, which is needed for the phase transition in MoO_3-x_.

The following equations show a possible hydrolysis reaction between water and MoO_3-x_ containing oxygen vacancy, based on literature^46^.

$${\text{MoO}}_{{3}} \;(\alpha) \to {\text{MoO}}_{{{3} - {\text{x}}}} \;(am)\quad ({\text{PVD}};\;{\text{bulk}} \to {\text{nano}}\;{\text{ size}},\;{\text{Implantation }}\;{\text{of}}\;{\text{Vo}}^{{\text{i}}} \;{\text{defect}})$$

$${\text{2MoO}}_{{{3} - {\text{x}}}} \left( {am} \right) \to {\text{2MoO}}_{{{3} - ({\text{x}} + {\text{y}})}}^{{{\text{n}} + }} + { 1/2}\;{\text{ O}}_{{2}} \quad ({\text{VST}};\;{\text{ Implantation }}\;{\text{of}}\;{\text{Vo}}^{{{\text{ii}}}} \;{\text{defect}},\;{\text{at 6}}0{-}{9}0\;^{ \circ } {\text{C/vacuum}})$$

$${\text{2MoO}}_{{{2}.{5}}}^{{{1} + }} + {\text{ H}}_{{2}} {\text{O}} \leftrightarrow {\text{MoO}}_{{4}}^{{{2} - }} + {\text{ MoO}}_{{2}} + {\text{ 2H}}^{{ + }{}} \quad ({\text{K}}_{{\text{a}}} = {1 } \times { 1}0^{{ - {14}}})$$

$${\text{MoO}}_{{4}}^{{{2} - }} + {\text{ 2H}}^{ + } \leftrightarrow {\text{ H}}_{{2}} {\text{MoO}}_{{4}}$$

$${\text{H}}_{{2}} {\text{MoO}}_{{4}} \to {\text{MoO}}_{{3}} \;\left( {h \, \;{\text{or}}\;\alpha } \right) + {\text{ H}}_{{2}} {\text{O}}$$

$${\text{MoO}}_{{2}} + {\text{ 1/2 O}}_{{2}} \to {\text{MoO}}_{{3}} \;\left( {h \, \;{\text{or}}\;\alpha } \right)$$

According to the above equations, the commercially acquired bulk *α*-MoO_3_ material was initially converted into an amorphous (*am*-) MoO_3-x_ nanomaterial, with implanted primary oxygen vacancies (Vo^i^), at the initial PVD step. Then, these *am*-MoO_3-x_ nanomaterials were ultrasonically extracted (USE) and subjected to VST for implanting secondary oxygen vacancies (Vo^ii^), followed by the formation of a (MoO_3-(x+y)_)^n+^ cation. This cation immediately got stabilized itself by reacting with solvent environment (H_2_O) to form intermediates like MoO_2_ and molybdate anion (MoO_4_^2−^). Further this anion reacted with available protons to produce less stable molybdic acid followed by its decomposition into molybdenum trioxide, with a new crystal phase (hexagonal* h*-MoO_3_ or orthorhombic *α*-MoO_3_). The by-product MoO_2_ also reacted with left over oxygen atoms to produce MoO_3_ with new phase.

In the case of *iso*-propanol, it released protons slowly or produced lesser number of protons in the medium as its dissociation was weak. Hence, the solvation of (MoO_3-(x+y)_)^n+^ cation was also slow and weak. Because of the inadequate concentration of released protons (H^+^) in the medium, the hydrolysis of molybdate anions into molybdic acid, followed by its decomposition into *α*-MoO_3_ was only achieved partially according to the stoichiometric equations mentioned below. Therefore, the product was the mixed phases of *h* and *α*-MoO_3_, but dominated by *h*-phase as evidenced by XRD.

$${\text{MoO}}_{{3}} \;\left( \alpha \right) \to {\text{MoO}}_{{{3} - {\text{x}}}} \;\left( {am} \right)\quad ({\text{PVD}}; \, \;{\text{bulk}} \to {\text{nano }}\;{\text{size}}, \, \;{\text{Implantation}}\;{\text{ of}}\;{\text{Vo}}^{{\text{i}}} \;{\text{defect}})$$

$${\text{4MoO}}_{{{3} - {\text{x}}}} \;\left( {am} \right) \to {\text{4MoO}}_{{{3} - ({\text{x}} + {\text{y}})}}^{{{\text{n}} + }} + {\text{ O}}_{{2}} \quad ({\text{VST}};\;{\text{ Implantation }}\;{\text{of}}\;{\text{Vo}}^{{{\text{ii}}}} \;{\text{defect}},\;{\text{at 9}}0\;^{ \circ } {\text{C/vacuum}})$$

$${\text{4MoO}}_{{{2}.{5}}}^{{{1} + }} + {2}\left( {{\text{CH}}_{{3}} } \right)_{{2}} {\text{CHOH }} + {\text{O}}_{{2}} \leftrightarrow {\text{ 2MoO}}_{{4}}^{{{2} - }} + {\text{2MoO}}_{{2}} + {\text{4H}}^{ + } + {2}\left( {{\text{CH}}_{{3}} } \right)_{{2}} {\text{C }}{ =} {\text{ O}} \ldots \left( {{\text{K}}_{{\text{a}}} = { 1 } \times {1}0^{{ - {18}}} } \right)$$

$${\text{2MoO}}_{{4}}^{{{2} - }} + {\text{4H}}^{ + } \leftrightarrow {\text{2H}}_{{2}} {\text{MoO}}_{{4}}$$

$${\text{2H}}_{{2}} {\text{MoO}}_{{4}} \to {\text{2MoO}}_{{3}} \left( {h \, \;{\text{and}}\;\alpha } \right) + {\text{2H}}_{{2}} {\text{O}}$$

$${\text{2MoO}}_{{2}} + {\text{O}}_{{2}} \to {\text{2MoO}}_{{3}} \;\left( {h \, \;{\text{and}}\;\alpha } \right)$$

Therefore, the release of protons from solvent dissociation was the rate determining step in both the case of water and *iso*-propanol, which largely affected the intermediate and final product formation with phase transition.

$${\text{MoO}}_{{\text{3 - x}}} + {\text{ H}}_{{2}} {\text{O}}\mathop{\longrightarrow}\limits^{{ {6}0^\circ {\text{C}}}} h{\text{ - MoO}}_{{3}} \quad \quad \quad \quad \quad \quad \quad \quad\quad\left( {w60} \right)$$

$${\text{MoO}}_{{\text{3 - x}}} + {\text{ H}}_{{2}} {\text{O}}\mathop{\longrightarrow}\limits^{{ {9}0^\circ {\text{C}}}}\alpha {\text{ - MoO}}_{{3}} \quad \quad \quad \quad \quad \quad \quad \quad\quad\left( {w90} \right)$$

$${\text{MoO}}_{{\text{3 - x}}} + \, \left( {{\text{CH}}_{{3}} } \right)_{{2}} {\text{CHOH}}\mathop{\longrightarrow}\limits^{{ {9}0^\circ {\text{C}}}}h \, \;{\text{and}}\; \, \alpha {\text{ - MoO}}_{{3}} \quad \quad \quad \left( {ipa90} \right)$$

We realize that our reasoning behind solvent-mediated mechanism is only at theoretical level and not experimentally proven yet. To negate this shortcoming, we will be doing in-situ spectroscopic measurements to understand the stoichiometric chemistry, reaction kinetics, and mechanism of this phase transition, in our future studies. These experiments will reveal the nature and distribution of oxygen vacancies, nature of intermediates, solvation level, proton concentration, nucleation etc.

### Discerning the oxygen defects

Effective control and design of oxygen vacancy sites within the material are crucial for intended applications. There are some indirect indications for the presence of oxygen vacancies (Vo^i^ or Vo^ii^) in our synthesized materials (MO_w60_, MO_w90_, MO_ipa90_), as evidenced by spectroscopic characterizations. FT-IR spectra (Fig. 1) showed some peaks, due to single oxygen atom interaction with three molybdenum atoms (548 cm^−1^), Mo–O–Mo bonding with stretching vibrations of O_3_ atoms (864 cm^−1^), Mo–O vibrations (601 cm^−1^), and Mo_2_–O_4_ bonding by solvolyzing H_2_O molecules (466 cm^−1^). Similarly, Raman spectra of the samples also showed peaks for O–Mo–O bonds (300–700 cm^−1^) and Mo–O–Mo bonding (700–1000 cm^−1^). But again, these are all qualitative indications only.

Oxygen vacancies (Vo^i^, Vo^ii^), which alter the electronic environment at molecular level, is detectable, by observing changes in the binding energies of Mo-3d and O-1s peaks in XPS spectra too. Reduced oxidation states of Mo (from Mo^6+^ to Mo^5+^ or Mo^4+^) undergo shift in Mo-3d peaks, while the O-1s peaks undergo shift and change in shape as described in XPS data interpretation (Fig. 9, Table S5). However, quantitative analysis of these oxygen peaks only could reveal the concentration and distribution of oxygen vacancies, which is crucial for the interpretation of mechanism of phase transition.

Hence, we have quantified the area under the O-1s peak for all 3 samples (Fig. 9b, Table S5), which conveys that the thermodynamically most stable *α*-MoO_3_ obtained via w90 treatment possess more oxygen concentration than the metastable *h*-MoO_3_ obtained from w60, and *h* and *α*-MoO_3_ from ipa90 treatments. Table S5 shows that more the oxygen content, better the stability and phase transition. Because, the oxygen deficient and defective (MoO_3-(x+y)_)^n+^cation is hungry of oxygen atoms which has to be quenched by supplying fresh oxygen atoms and then be stabilized by protons, coming from either reagents or solvents. These results suggest that w90 treatment (solvolysis) could have efficiently refilled the oxygen vacancies in (MoO_3-(x+y)_)^n+^, by the incoming oxygen atoms from H_2_O solvent and protonation of MoO_4_^2−^ intermediate by the same H_2_O, with the complete phase transition (*am* → *h* → *α*-MoO_3_). Whereas in w60, ipa90 treatments (solvolysis), the substitution of oxygen atoms from solvents: H_2_O at 60 °C, *iso*-propanol at 90 °C, was not that efficient, to fill the oxygen vacancies and for the protonated stabilization of MoO_4_^2−^ intermediate. Hence, they were left with incomplete and partial transitions like, only up to *h*-MoO_3_ and mixed *h* and *α*-MoO_3_ respectively. So, it was purely because of elevated temperature (90 °C) and more protic solvent like H_2_O, the phase transition propelled from *h*- to *α-*crystalline structure. Thus, from XPS data perspective, it was inferred that MO_w60_, MO_ipa90_ samples possess more oxygen vacancies than MO_w90_, in which the vacancies were efficiently refilled.

The real limitation here is that the identification of source of oxygen atoms in *α*-MoO_3_ and *h*-MoO_3_ product phases. For this, we need an in-depth spectroscopic (Mass/IR) study of O^18^ isotope labelled-H_2_O^18^ mediated VST of MoO_3-x_. Another way of characterizing the oxygen vacancies would be using in-situ EPR spectroscopy to find out the nature of oxygen defected and then refilled. This way the exact nature and distribution of oxygen vacancies can be characterized. We keep this task for our future work and would definitely want to report the scientific community later.

Further, our strategy to control the oxygen defects was an indirect approach by measuring band gaps via UV–Vis spectroscopy. The principle is that introducing oxygen vacancies creates new energy levels and when their concentration exceeds 0.1%, significantly alters the energy bands in the material. In transition metal oxide semiconductors, oxygen vacancies modify the valence band, which consists mainly of the oxygen 2p orbital, that leads to changes in the material's optical absorption properties^47^. Yuan et al*.* studied how different calcination rates for metal oxides influence the number of oxygen vacancy sites. Their findings suggest that increasing oxygen vacancies can reduce the band gap value of the material^48^.

In our case, Fig. 3b, showcases the determination of band gaps (E_g_) through Tauc plot analysis using the UV–vis spectrum. The calculated E_g_ values for MO_w60_ (*h*-MoO_3_), MO_w90_ (*α*-MoO_3_), and MO_ipa90_ (*h* and *α*-MoO_3_) were 2.94 eV, 2.45 eV, and 1.51 eV respectively. Based on above mentioned Yuan et al's postulate, the total oxygen vacancies (Vo = Vo^i^ + Vo^ii^) in our samples could be in the order for; MO_ipa90_ > MO_w90_ > MO_w60_. This indicated that organic solvent like *iso*-propanol (ipa90) medium could have created more secondary oxygen vacancies (Vo^ii^) than an aqueous solvent (H_2_O) medium (w90), followed by w60 medium, during VST. However, the solvolysis efficiency of refilling oxygen in the vacancy sites followed by proton mediated stabilization of MoO_4_^2−^ intermediate was better at 90 °C, especially with H_2_O. Hence the degree of phase transition to *α*- phase, in our samples, was in the reverse order for; MO_w90_ > MO_ipa90_ > MO_w60_. At 60 °C in H_2_O, both the Vo created as well as the solvolysis were less and inefficient. Thus, MO_w60_ ended up with phase transition (*am → h*) only up to metastable hexagonal phase (*h*), whereas MO_w90_ resulted in complete phase transition from *am-* to *h*-, then to *α*-MoO_3_, and finally MO_ipa90_ yielding only partial transition (mixed phases of *h* and *α*-MoO_3_). Here comes a disagreement between, XPS and UV–Vis data for MO_w60_ sample, in which O-1s area suggested the presence of more Vo, whereas E_g_ value indicated less Vo in it. It may be because of the less expression of oxygen atoms from the meta stable *h*-MoO_3_ (MO_w60_) in XPS causing less sensitivity towards oxygen atoms. We will sort out this discrepancy in our future work.

Thus, oxygen defects can be correlated to bang gaps (E_g_) and vice-versa via UV–Vis spectroscopy. Therefore, the E_g_ values are of one kind of indirect indication for us, to change the synthesis parameters and have a control over the distribution and concentration of oxygen defects within the crystal lattice. This was our strategy to control the oxygen vacancies in quantitative terms.

Others in reported methods, often employed high temperature annealing treatment in a reducing atmosphere to generate oxygen vacancies in metal oxide materials. The concentration of these vacancies can be controlled to some extent by adjusting parameters such as temperature, vacuum level, atmosphere composition, inert gas and other process variables. But we have controlled it through simple vacuum assisted solvolysis technique (VST) in a cost-effective way, in our present study. Thus, we believed that applying a controlled heat under vacuum and then neutralizing the positively charged defective MoO_3_ using protic solvents, could yield desired crystalline phase transition in our materials. This was the hypothesis behind our experimental planning and execution.

### Repeatability and reproducibility of *DEST* method for an eco-friendly, energy efficient, low temperature phase transition in MoO_3_

Wet-chemical syntheses would often result in issues like reproducibility and heterogeneity in the sample. We have carefully avoided these issues as much as possible, by paying more attention on exact repetition of operation parameters (precursor weight, temperature, time, vacuum level, solvent quantities) in multiple synthesis trials to confirm the repeatability and reproducibility (Fig. S3). SEM (Fig. 7a–c) showed the homogeneity within each sample in terms of particle size and morphological appearance.

Currently we rely on the microscopic techniques and XRD to address sample heterogeneity if anything of that sort found. For e.g. The Rietveld analysis of the X-ray diffraction pattern of MO_ipa90_ confirms the presence of (010) of *h*-MoO_3_ and #(020) of *α*-MoO_3_ phases, within the same sample (Fig. 4c). This indicates the heterogeneity and composition of mixed phases, but predominately composed of* h*-MoO_3_. The software reveals that the mixed phase contains 72.6% of *h-*MoO_3_ and 27.4% of *α*-MoO_3_. This ratio can be controlled by tuning the solvent amount, mixtures of solvents, ratios of solvents, material concentration etc. It is good to maintain the homogeneity and avoid heterogeneity in the samples prepared. But sometimes heterogeneity could also give us unexpected positive turn around in applications, due to synergy between multiple phases. So, it is not always undesirable to have heterogeneity in the synthesized samples.

Figure 4 and Fig. S3 show the XRD spectra of samples from original batch (Batch 1) and reproduced materials (Batch 2) respectively. Table S6 shows the comparison of X-Ray diffraction angles (2θ) between two batches of samples. The results suggest that careful repetition of synthesis parameters (temperature, time, vacuum level, solvent volume, precursor amount) yielded same materials as the 2θ values were almost similar, except few fractions of degree deviation, here and there. Hence, this *DEST* synthesis protocol is reproducible. Thus, we believe that our experimental design is reliable and has clear control over all the variables involved in the products’ formation.

### Discrepancies between our findings and reported results

Tables S7, S8 show how our method of executing phase transition in MoO_3_ differs from other previously reported methods. Basically, our method doesn’t require very high temperature for phase transition and also employs facile procedure with eco-friendly low boiling solvents, as against the phase transitions which are often carried out at high temperatures (150–450 °C), that consumes lot of thermal energy and energy costs.

It is widely reported that amorphous (non-crystalline/nano) materials are always good starting points for the formation of phase-pure materials of specific mono-morph, through controlled crystallization^49^. Our work involves in converting amorphous MoO_3-x_ into* h*-MoO_3_, *α*-MoO_3_ and mixed phases (*h *and* α*) at the maximum 60–90 °C using either water or *iso*-propanol in a simple vacuum distillation unit. However, the reported methods start with crystalline precursor materials like NH_4_^+^, Na^+^ cation containing molybdate, heptamolybdate compounds. These cations play a catalytic/promotor role in nucleation and crystal growth steps of various forms of MoO_3_, which is not the case in our method. Other major difference is that they use non-eco-friendly, stochiometric reagents like highly corrosive, strong mineral acids e.g., HCl, HNO_3_ etc., in order to hydrolyze molybdate and heptamolybdate feed molecules, during hydrothermal syntheses. They also report that variety of crystalline phases can be formed at different temperatures and various ratios of precursor to acid solvent. But some of their lattice parameters^21,35^ and band gaps have notable changes from our values, for the given crystal structure. This comparative preparation and phase transition conditions for MoO_3_ declares that our method is better than the reported methods. Thus, we defend our work, as a reasonable contribution to nanomaterials synthesis field.

The discrepancies between our method and reported methods can throw up new application possibilities in electrochemistry, energy storage, photocatalysis, solar cells, adsorbents, etc., as every method produce a material with unique properties such as lattice parameters, band gap, interlayer spacing etc. This also has lot of scope for extensive characterization of these materials which can impact overall trajectory of materials science research.

### Electrochemical investigations of *DEST*-made MoO_3_ polymorph 2D nanosheet materials

Any material in the universe should justify its existence for an application. Hence, we have investigated the synthesized materials for supercapacitor performance, which is our model application study. Though it is proclaimed that *α*-MoO_3_ is the most electroactive phase, still we wanted to have a comparison with *h*-MoO_3_ and mixed phase (*h* and *α*-MoO_3_), as they were prepared from new synthetic route. We never know what are all happening behind in an unknown synthesis method. So, it is good to make an attempt and see the results to have better confirmation.

**Figure 10:**
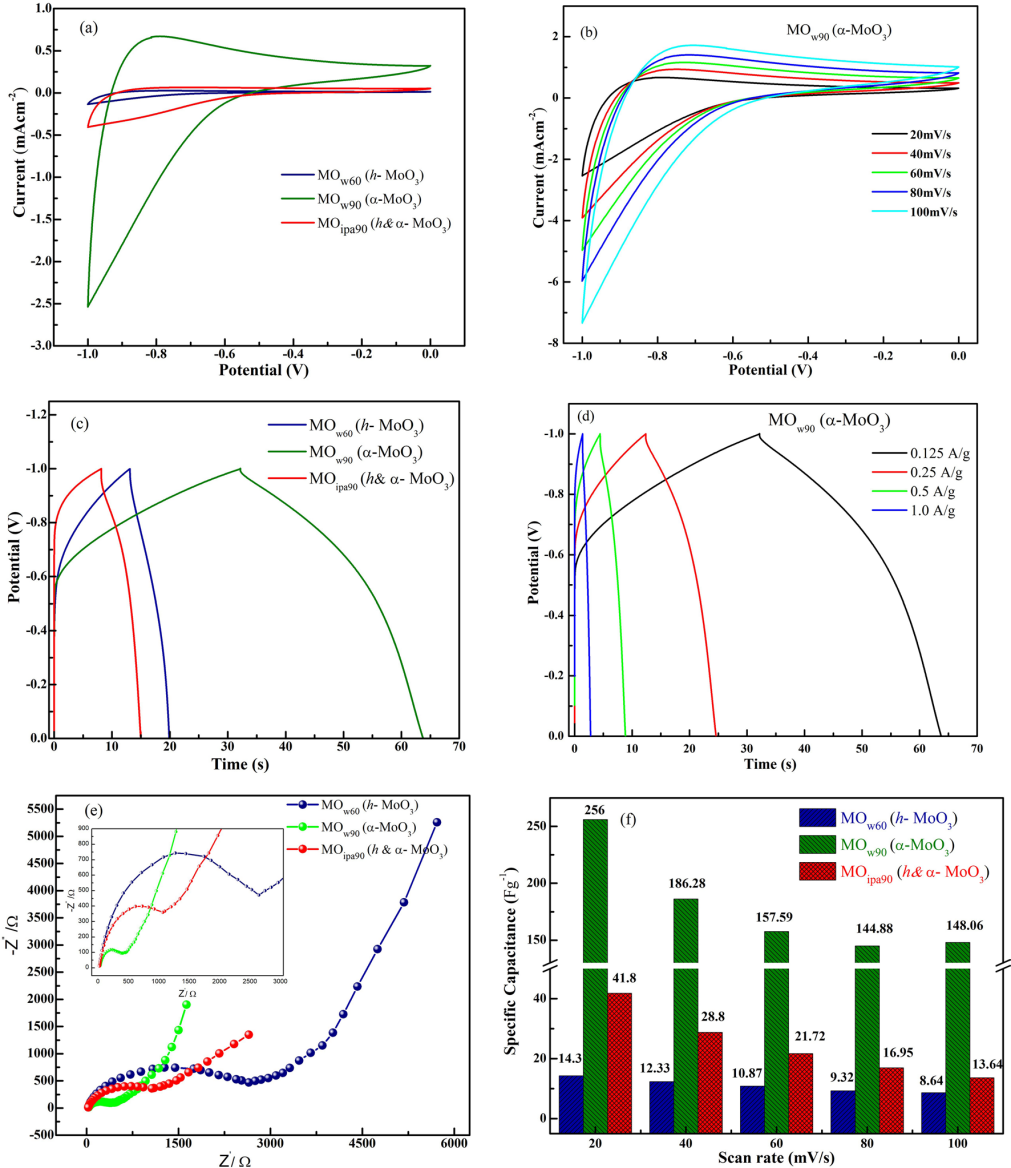
Electrochemical performances of *DEST*-made MoO_3_ polymorphs modified glassy carbon electrodes (GCE): (**a**) Cyclic Voltammetric (CV) curves for all polymorphs at the scan rate of 20 mV s^−1^, (**b**) CVs at different scan rates for *α* -MoO_3_ (MO_w90_), (**c**) Galvanostatic Charging-Discharging (GCD) characterization for all polymorphs at the current density of 0.125 A/g, (**d**) GCD profiles at different current densities for *α* -MoO_3_ (MO_w90_), (**e**) Electrochemical impedance spectroscopy (EIS) for all polymorphs, (**f**) Specific capacitance values of all polymorphs at different scan rate.

The electrochemical performance of *DEST*-made MoO_3_ polymorphs modified glassy carbon electrodes (GCE) were examined by cyclic voltammetry (CV), galvanostatic charge–discharge (GCD) and electrochemical impedance spectroscopy (EIS) measurements. There was no leaching of loaded material from GCE surface observed, in entirety of experiments. The area under the curve in CV (Fig. 10a) for *α*-MoO_3_ (MO_w90_) was significantly greater than that of other polymorphs; *h*-MoO_3_ and mixed *h* and *α*-MoO_3_ (MO_w60_, MO_ipa90_) at the scan rate of 20 mV s^−1^, showing significantly increased charge/power density. Figure 10b, showing the CV curves exclusively for the best performing *α*-MoO_3_ (MO_w90_), recorded in the potential range, − 1.0 to 0 V and at different scan rates starting from 20 to 100 mV/s. The area under the curve increased when the scan rate increased and all CV curves exhibited quasi-rectangular shapes without any redox peaks, indicating the electrochemical stability of the material.

Further, galvanostatic charge–discharge curves (GCD) were recorded at current density, 0.125 A/g, to assess the capacitance behavior of MoO_3_ polymorphs, *h*-MoO_3_ (MO_w60_), *α*-MoO_3_ (MO_w90_) and *h* and *α*-MoO_3_ (MO_ipa90_), as shown Fig. 10c. All three MoO_3_ polymorphs were displaying a non-linear charge–discharge curve, demonstrating their pseudocapacitive nature. As expected, *α*-MoO_3_ (MO_w90_) showed slow and steady pace of charging and discharging, effectively suggesting the superior capacitance behavior than that of other polymorphs (based on the Eq. 1 in ‘Methods' section). GCD measurements were also done at different current densities for the best performing *α*-MoO_3_ (MO_w90_) as shown in Fig. 10d. Current densities higher than 0.125 A/g was found to be detrimental in terms of capacitance measurement, thus suggesting 0.125 A/g was the optimum current density for the *DEST*-made MoO_3_ polymorphs.

Electrochemical impedance spectroscopy (EIS) experiment was conducted in order to learn more about the electrocapacitive behavior of the aforementioned MoO_3_ polymorphs. Figure 10e displays the Nyquist plot for each sample. All three samples showed a no electrode resistance, but electrolyte resistance. The impedance response in the Nyquist plot can be split into two regions, (i) an initial semi-circular electrolyte resistance region, and further (ii) an upward straight line of resistance free region. The semicircle directly indicates the initial resistance that the material experiences, which is in the order for *h*-MoO_3_ (MO_w60_) > *h* and *α*-MoO_3_ (MO_ipa90_) > *α*-MoO_3_ (MO_w90_). Thus, confirming the superior conducting and capacitive nature of *α*-MoO_3_ (MO_w90_) over other polymorphs.

Figure 10f shows the calculated specific capacitance values at different scan rates for all three MoO_3_ polymorphs. Based on the calculations using Eq. (1) and Fig. 10f, the specific capacitance for MO_w60_ (*h*-MoO_3_), MO_w90_ (*α*-MoO_3_) and MO_ipa90_ (*h *and* α*-MoO_3_) were found to be 14.3 Fg^−1^, 256 Fg^−1^, 41.8 Fg^−1^ respectively. Thus, MO_w90_ (*α*-MoO_3_) proved to be having 18 times better capacitance than its sister material MO_w60_ (*h*-MoO_3_) and 6 times better performing than its counterpart MO_ipa90_ (*h *and* α*-MoO_3_). This result is at par excellence than other reported materials in the literature. The comparative performance analysis with existing *α*-MoO_3_ and non-MoO_3_ coated electrode materials in literature, is furnished in Tables S9, S10. Similar to earlier mentioned discrepancies in lattice parameters and band gaps, based on preparation method and phase transition temperature (Tables S7, S8), the reported capacitance values for *α*-MoO_3_ also differs from our value (256 Fg^−1^), but largely in decrement. It shows that the reported *α*-MoO_3_ materials, where the phase transition was achieved only above 400 °C, exhibit specific capacitance, only below 200 Fg^−1^. The electrode materials other than MoO_3_ (non-MoO_3_) also show similar trend i.e., < 200 Fg^−1^. Thus, our material is competitive and promising, comparatively with other reported MoO_3_ materials and conducting polymers.

### Structure–activity relationship

#### Role of oxygen defects

Oxygen vacancies (Vo), which are the predominant point defects in metal oxides, have the tendency to increase the electron density in the local environment of the molecular structure. This increase is caused by electrons donated by the escaped oxygen atoms, leading to an improved electrical conductivity and catalytic performance of the product material. e.g., particularly in n-type MoO_3_ semiconductors^50^. Oxygen vacancies (Vo) can also significantly increase the interlayer spacing because of loss of oxygen atoms. This also enhances the electrochemical activity and thereby promoting faster charge storage kinetics^51^. For example, annealing in a reducing environment can increase oxygen vacancy concentration, improving lithium–ion intercalation kinetics in battery applications. Thus, the control over oxygen defect formation can result in desired electrochemical properties such as conductivity, capacitance etc. in the target material. In our case, MO_ipa90_ (*h* and *α*-MoO_3_) sample has more oxygen vacancies (Vo) than MO_w90_ (*α*-MoO_3_) sample, based on the E_g_ values for MO_w60_ (*h*-MoO_3_), MO_w90_ (*α*-MoO_3_), and MO_ipa90_ (*h* and *α*-MoO_3_) ; 2.94 eV, 2.45 eV, and 1.51 eV respectively (Vo: MO_ipa90_ > MO_w90_ > MO_w60_). However, the electro-capacitance activity was higher (256 Fg^−1^) with MO_w90_ sample than MO_ipa90_ sample (41.8 Fg^−1^). Hence, this suggests that more than oxygen vacancies, the lattice parameters in the crystalline phase and interlayer spacing were playing a major role in electrochemical responses of the MoO_3_ polymorphs.

#### Role of crystal characteristics

Characteristics like lattice parameters, density of states, surface area, and morphology vary for different crystal structures of the same compound. Lattice parameters influence ion intercalation and diffusion pathways, whereas the density of states near the Fermi level impacts electronic conductivity and charge storage. Surface and morphological features like area, roughness and defects affect the availability of catalytic active sites for charge storage. Hence, the overall crystal structure influences electrochemical properties consequently, in large.

**Figure 11:**
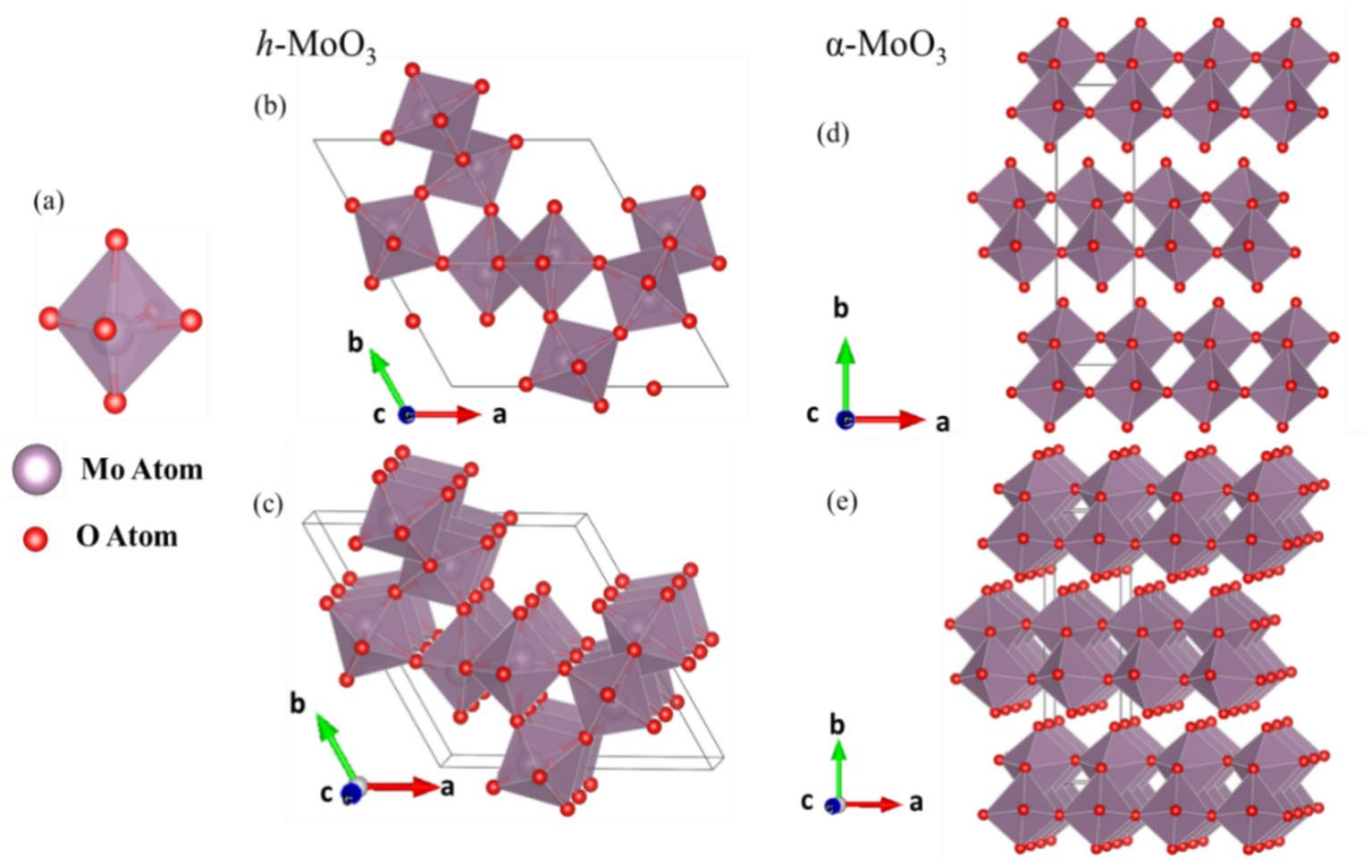
A schematic presentation of MoO_6_ octahedron (**a**), composed of Mo and O atoms, forming MoO_3_ crystal structures; *h* -MoO_3_ (**b**,**c**), *α* -MoO_3_ (**d**,**e**). The unit cell dimensions are denoted by the grey lines.

MoO_3_ exists in polymorphs, such as *h*-MoO_3_ (hexagonal), *α*-MoO_3_ (orthorhombic), *β*-MoO_3_ (monoclinic), and *γ*-MoO_3_ (cubic)^4–7^. The structure of different phases of MoO_3_ is established by how the foundational octahedral unit (MoO_6_) shares its corners and edges for ions’ interactions^10^. The configuration of MoO_6_ octahedrons (Fig. 11a) is influenced by external factors including temperature, pressure, and impurities^52^. The *β*-MoO_3_ structure (Fig. 5b) is made up of MoO_6_ octahedra that share corners in three dimensions and thus creating a monoclinic structure. It is similar to the structure of WO_3_ and related to distorted ReO_3_ type structure, thus classified as an empty A-site perovskite (ABO_3_). *β*-MoO_3_ holds only corner sharing oxygen atoms, whereas *h-*MoO_3_ and *α*-MoO_3_ having both corner and edge sharing oxygen atoms. The *h-*MoO_3_ is constructed by zigzag chains of same MoO_6_ octahedra (Fig. 11b,c), along [001] direction, which creates massive 1D tunnels. These chains are interlinked between them at *cis*-position. Whereas, *α*-MoO_3_ is composed with edge-sharing zigzag rows and corner-sharing rows of deformed MoO_6_ octahedra along the [001] and [100] directions respectively, to form a planar double layer (2D), giving rise to an anisotropic structure. Strong covalent and ionic bonds dominate the internal interactions in the deformed MoO_6_ octahedra, which are kept together in the vertical [010] direction by weak van der Waals' forces, that causes the stratification in* α*-MoO_3_. Double layers of MoO_6_ octahedra are arranged in a (ABA) pattern and repeated in the *ab* plane along the *b* axis to produce a layered framework in the crystal structure of *α*-MoO_3_^9,26^ (Fig. 11d,e). These structural differences between various crystalline phases can have influence over the electrochemical properties of MoO_3_ polymorphs.

Among the MoO_3_ polymorphs, *α*-MoO_3_ generally provides the highest specific capacitance due to its thermodynamic stability and favorable layered structure that facilitates ion intercalation and de-intercalation^11,15^. However, the actual performance can also be influenced by other factors such as synthesis methods, morphology, particle size, thickness of the nanosheets, lattice parameters, interlayer spacing etc. In our case (Table 1), the *α*-MoO_3_ (MO_w90_) possess smaller lattice parameters (a = 3.95, b = 3.95, c = 3.72) than *h-*MoO_3_ (a = 10.61, b = 10.61, c = 3.72), and an enhanced interlayer spacing of 3.8 Å (101). This could have resulted more surface area in *α-* MoO_3_, than *h-*MoO_3_ (MO_w60_), as *α*-MoO_3_ was derived from the intermediate (MoO_3-(x+y)_)^n+^ having more secondary oxygen vacancies ( Vo^ii^), caused by w90 treatment in VST. It is also believed that orthorhombic (*α*-) structure possess high fraction of surface exposed atoms^53,54^ than hexagonal structure (*h*-), due to smaller lattice and enhanced interlayer spacing. Hence it could have more active sites for charge storage. Thus, these structural differences between the metastable *h-*MoO_3_ and thermodynamically stable *α*-MoO_3_ have their direct influence on the capacitance values; former perfoms (14.3 Fg^−1^) poorer than latter (256 Fg^−1^), whereas the mixed phase, performs moderately (41.8 Fg^−1^).

**Table 1:** Comparison of crucial characterization data of MoO_3_ polymorphs to discern their electrochemical activity.

Structure–activity relationship (SAR)									
S. no.	Sample ID	XRD			HRTEM		UV–Vis	XPS	CV
		Lattice parameters			Interplanar spacing (d) Å		Band gap (E_g_)	O-1s peak area (× 10^4^)	Specific capacitance C_*p*_ F/g
		*a*	*b*	*c*					
1	MO_w60_ (*h*-MoO_3_)	10.61	10.61	3.72	(011)	3.47	2.94	20.93	14.3
					(120)	3.40			
					(030)	3.05			
2	MO_w90_ (*α*-MoO_3_)	3.95	3.95	3.72	(210)	3.2	2.45	42.76	256
					(101)	3.8			
3	MO_ipa90_ (Mixed Phases of 72.6% *h*-MoO_3_ and 27.4% *α*-MoO_3_)	10.61/3.98	10.61/13.86	3.72/3.71	(501)	2.2	1.5	24.92	41.8
					(121)	2.5			

We were expecting a synergistic effect from the mixed-phase MoO_3_ (*h* and *α*) viz-a-viz its electrochemical performance. But the result did not meet our expectation. The mixed phase usually causes variations in the local chemical environment, including differences in stoichiometry, surface defects, oxidation states, adsorbed species, and bonding configurations. These variations affect the electron density around Mo atoms. We also believe that there might be some unknown interfacial sites which could have distorted lattice parameters and modified density of states near the Fermi level. These interfaces at the phase boundaries can influence ion diffusion pathways and charge transfer, which will have an overall impact on electronic conductivity, charging-discharging ability of the material. To experimentally prove this interfacial phenomenon, pure *h*-MoO_3_ and pure *α*-MoO_3_ crystalline phases can be physically mixed in various known ratios and then be characterized by UV–Vis, XRD, XPS, CV, GCD. This might offer a preliminary understanding of how the bulk level-interface influence the electrochemical behaviour of the mixed phase. Based on this, MoO_3_ polymorphs materials possesing lattice level-interfaces can be synthesized and further scrutinized by Rietveld refinement technique in XRD using FULLPROF program.

These new lattice level-interfaces can be created by tuning the synthesis parameters especially the solvents. We realize that the current ratio (72.6% of *h-*MoO_3_ and 27.4% of *α*-MoO_3_) in the mixed phase was not favorable for enhanced capacitance. Hence, for e.g. a synthesis of (50:50) of *h-*MoO_3_:* α*-MoO_3_ or a more of* α*-MoO_3_ and less of *h-*MoO_3_ combination (75:25, 60:40), could help in achieving the desired results, as we already know that *α*-MoO_3_ is relatively more electro-active and capacitive. We believe that this ratio can be adjusted by employing different organic solvents (ethanol, acetic acid etc.) for the creation of defective (MoO_3-(x+y)_)^n+^ and its subsequent hydrolysis with different degrees of phase transition. This way a final product of lattice level-mixture of various ratios of MoO_3_ polymorphs can be obtained. This study could enable the most anticipated synergistic effect from the mixed-phase MoO_3_ (*h* and *α*) for its enhanced electrochemical performance.

One interesting point to be noted here is that, even though the interlayer spacing in mixed-phase MoO_3_ (*h* and *α*) was lower (2.5 Å) than *h*-MoO_3_ (MO_w60_) (3.47 Å) (Table 1), it was more electro-active than* h*-MoO_3_. So, there is definitely a scope for enhancing its capacitance further through synergistic effect, given right preparation conditions are used. We keep this more nuanced work for our future activities.

### Factors affecting electrochemical behavior

Based on the results and literature evidences, the factors which might largely affect the electrochemical behavior of MoO_3_ are (a) synthesis methods, (b) oxygen vacancies, (c) crystal structure and interfaces, (d) morphology, (e) particle size, (f) thickness of the nanosheets, (g) interlayer spacing etc. However, the actual performance can also be influenced by factors other than these material properties; for e.g., specific conditions of electrochemical testing such as (a) amount of material loading on GCE, (b) choice of electrolyte, (c) electrodes’ configuration etc. Optimizing all these factors can enhance the specific capacitance of each mono-morph to the desired performance level. We will look into these details very elaborately in our future work.

### Future prospects

Apart from the proposed future courses of actions mentioned in the discussion part, we would also like to scale up of the production of MoO_3_ and studying its long-term stability through longer hours experiments (charging-discharging), performing multiple cycles of GCD. We are excited to report our findings in our next communication.

## Conclusion

In summary, physical vapor deposited (PV**D**) amorphous MoO_3-x_ nano powder was meticulously extracted by ultrasonication (US**E**) in strategically chosen green solvents. Further, the extracted MoO_3-x_ nanomaterials were recrystallized by an eco-friendly, energy efficient, vacuum assisted solvothermal (VS**T**) approach at very low temperature in water or *iso*-propanol. The combination of operating parameters in this *DEST* synthesis has truly yielded *h*-MoO_3_ even at 60 °C and a fascinating phase transition from *h*-MoO_3_ to *α*-MoO_3_ at very low temperature *ca.* 90 °C, in just water. On the other hand, *iso*-propanol as a solvent, produced mixed phases (*h *and* α*) of MoO_3_ at 90 °C. The different phases of synthesized MoO_3_ were identified and confirmed by an extensive XRD spectroscopic scrutiny and other characterizations. We believe that the implantation of oxygen defects followed by its solvolysis with the protic nature of the solvent and optimally very low temperature selection, resulted in an efficient proton mediated phase transition (*am → h → α*-MoO_3_) in first-of-its kind. Because, other reported methods have always used very high calcination temperature ranges (300–1500 °C) to achieve this phase transition in MoO_3_.

To utilize the synthesized 2D nanosheets of MoO_3_ polymorph materials in real world application, a series of electrochemical investigations have been carried out under CV, GCD conditions. MO_w90_ (*α*-MoO_3_) found to be exhibiting 18 times better specific capacitance performance than MO_w60_ (*h*-MoO_3_) and six fold better activity than MO_ipa90_ (*h *and* α*-MoO_3_). We reasoned out that, *α*-MoO_3_ (MO_w90_) possess smaller lattice parameters and an enhanced interlayer spacing, which could have resulted more surface area and high fraction of surface exposed atoms in it, than *h-*MoO_3_ (MO_w60_), leading to more active sites for charge storage. These results convey the message that, by finetuning the synthesis parameters, electrochemical conditions further, *DEST* produced *α*-MoO_3_ 2D nanosheets can be made as the most promising upcoming candidates for creating next generation high-performance supercapacitors.

## Experimental

### Materials

Molybdenum Trioxide Extra Pure (MoO_3_) and *iso*-propanol ((CH_3_)_2_CHOH) were purchased from Loba Chemie. For the physical vapor deposition of MoO_3_ precursor the thermal evaporation BC 300 box coater was used. Milli-Q water was used in entire experiment.

## Methods

### Preparation of molybdenum trioxide (MoO_3-x_) thin film by physical vapor deposition (PVD)

Physical vapor deposition (PVD) of commercially acquired MoO_3_ powder (bulk material) was performed by using a less-expensive, homemade thermal box coater equipment. The glass substrates were cleaned before use, through Radio Corporation of America (RCA) procedure to remove organic and inorganic impurities settled on it^55^, followed by UV treatment to make the substrate’s surface hydrophilic^56,57^. Then the target material, molybdenum trioxide powder was taken and placed on the molybdenum boat in the thermal-ultrahigh vacuum evaporation set-up. MoO_3_ powder was evaporated at 700 °C and slowly deposited as thin films, layer by layer on the cleaned glass substrates, under the chamber pressure of 10^−7^ Pa. The thickness of the MoO_3_ films were controlled to be 150 nm by real time quartz crystal thickness monitor with constant rate of deposition ca. 0.3 nm/s^58,59^. Thus, the thin films of amorphous MoO_3-x_ materials were achieved (Fig. S4).

### Collection of amorphous MoO_3-x_ by ultrasonic extraction (USE)

The extraction of PVD-made MoO_3-x_ thin films was carried out using lab ultrasonic bath (USE). The MoO_3-x_ thin film coated glass substrates were immersed in two different polar solvents; water (H_2_O) and *iso*-propanol ((CH_3_)_2_CHOH), separately, to disperse the amorphous MoO_3-x_ flakes in respective solvents, by the application of sonication treatment for one hour. Thus, MoO_3-x_ thin films were peeled off carefully from the glass substrates in the form of nano powder and dissolved in above mentioned polar solvents. This led to get two transparent, homogeneous MoO_3-x_ solutions, separately (Fig. S4).

### Recrystallization of MoO_3-x_ by vacuum assisted solvothermal treatment (VST)

Further, the above aqueous and alcoholic MoO_3-x_ solutions were subjected to vacuum assisted solvothermal treatments (VST) using lab rotavapor equipment. The rotary evaporator set up basically had MoO_3-x_ solution containing rotating round bottom (RB) flask heated by metallic water bath filled with Milli-Q water and a chiller unit maintained at 7 °C to condense the extracted solvent. The aqueous and alcoholic MoO_3-x_ solutions were heated at different temperatures (60, 90 °C and 90 °C respectively) under constant 120 mbar vacuum, with the rotation speed of 175 rpm. The process was run continuously until entire solvent got evaporated with the outcome of MoO_3-x_ recrystallization (Fig. S4). At the end, three different colors of MoO_3_ (greyish green, blue and black) wet flakes were obtained for the respective treatments. These flakes were further vacuum dried in an oven at 60 °C to obtain the rigid solid flakes of (*h-*)*,* (*α-*)*,* (*h* and *α-*) MoO_3_ materials, correspondingly.

We have marked the *DEST*-made MoO_3_ samples as MO_w60_ (recrystallized from water at 60 °C), MO_w90_ (recrystallized from water at 90 °C) and MO_ipa90_ (recrystallized from *iso*-propanol at 90 °C).

### Characterization

Fourier Transform Infra-Red (FT-IR) Spectroscopy analyses of *DEST*-made MoO_3_ crystalline samples were carried out by Shimadzu Affinity S2, using KBr pellet method, in the wavenumber range of 500–4000 cm^−1^. Confocal Raman Spectroscopy of the samples was carried out by using WiTec Alpha 300 Germany equipment. Ultraviolet (UV)–Visible spectra of samples were recorded using Jasco V-770 Spectrophotometer. X-Ray Diffraction (XRD) patterns of samples were collected using PANalytical X-Ray Diffractometer, in the range of 2θ = 10° to 80°. The XRD patterns were simulated using the PCW software, utilizing crystallographic parameters such as space group, lattice parameters, and atomic positions from the literature. High Resolution Transmission Electron Microscopic (HRTEM) analysis was carried out using JEOL JEM 2100 microscope. Scanning electron microscopy (SEM) was performed by using Carl ZEISS EVO 18-Germany equipment with AMETEK Team V.4.3 EDS detector. Atomic Force Microscopy (AFM) was carried out using CS Instrument of Model No: S-SPM-00001. X-ray photoelectron spectra (XPS) were performed using K ALPHA + Thermo Fisher Scientific Instrument.

Electrochemical characterizations of all samples were carried out by M/S Biologic Science electrochemical workstation with a standard three-electrode cell configuration. The reference electrode was a standard Ag/AgCl, the counter electrode was Pt wire, and the working electrode was glassy carbon electrode (GCE). Initially, 2 mg of 2D MoO_3_ was dispersed in 500 µl of a polar solvent. Subsequently, 5 µl of this dispersion was applied on the GCE, by drop-casting technique, to modify its surface and as a fabrication of supercapacitor. An aqueous solution of 1 M Na_2_SO_4_ was used as the electrolyte, throughout the experiments. The electrochemical performance of different MoO_3_ polymorphs materials and the supercapacitor application was evaluated using cyclic voltammetry (CV) and galvanostatic charge–discharge method (GCD) respectively.

Equation (1) was used to calculate the specific capacitance from CV curves.

1 $$specific\; capacitance = C{\text{s}} = \frac{{{ }\int {{\text{i }}\;{\text{dV}}} }}{2m\;\Delta V \times scan\; rate }$$

Cs: specific capacitance, idV: integral part that represents the area below the cyclic voltammetry curve, m: mass of drop casted electrode material, ΔV: potential window.

## Supplementary Information

Supplementary Information.

## Data Availability

The data that support the findings of this study are available from the corresponding author upon request.

## References

[1] Liang, R. *et al.* Transition metal oxide electrode materials for supercapacitors: A review of recent developments. *Nanomaterials* **11**, 1248 (2021). 34068548 10.3390/nano11051248 PMC8151924

[2] Wan, Y. *et al.* Oxygen-deficient metal oxides for supercapacitive energy storage: From theoretical calculation to structural regulation and utilization. *Adv. Energy Sustain. Res.* **3**, 2100221 (2022). 10.1002/aesr.202100221

[3] Kalantar-Zadeh, K. *et al.* Synthesis of nanometre-thick MoO_3_ sheets. *Nanoscale* **2**, 429–433 (2010). 20644828 10.1039/B9NR00320G

[4] Muthamizh, S., Sengottaiyan, C., Jayavel, R. & Narayanan, V. Facile synthesis of phase tunable MoO_3_ nanostructures and their electrochemical sensing properties. *J. Nanosci. Nanotechnol.* **20**, 2823–2831 (2020). 31635618 10.1166/jnn.2020.17456

[5] Ren, H., Sun, S., Cui, J. & Li, X. Synthesis, functional modifications, and diversified applications of molybdenum oxides micro-/nanocrystals: A review. *Crystal Growth Des.* **18**, 6326–6369 (2018). 10.1021/acs.cgd.8b00894

[6] Etman, A. S. *et al.* Facile water-based strategy for synthesizing MoO_3–x_ nanosheets: Efficient visible light photocatalysts for dye degradation. *ACS Omega* **3**, 2193–2201 (2018). 31458524 10.1021/acsomega.8b00012 PMC6641438

[7] Paraguay-Delgado, F. *et al.* h-MoO_3_ phase transformation by four thermal analysis techniques. *J. Therm. Anal. Calorim.* **140**, 735–741 (2020). 10.1007/s10973-019-08842-0

[8] Chen, X., de Boer, R. M., Kosari, A., van Gog, H. & van Huis, M. A. Thermal reduction of MoO_3_ particles and formation of MoO_2_ nanosheets monitored by in situ transmission electron microscopy. *J. Phys. Chem. C* **127**, 21387–21398 (2023). 10.1021/acs.jpcc.3c05159 PMC10626599 37937158

[9] De Castro, I. A. *et al.* Molybdenum oxides—From fundamentals to functionality. *Adv. Mater.* **29**, 1701619 (2017). 10.1002/adma.201701619 28815807

[10] Zheng, L., Xu, Y., Jin, D. & Xie, Y. Novel metastable hexagonal MoO_3_ nanobelts: Synthesis, photochromic, and electrochromic properties. *Chem. Mater.* **21**, 5681–5690 (2009). 10.1021/cm9023887

[11] Sharma, R., Sharma, A. K., Jha, R. & Sarkar, A. Stable α-MoO_3_ nanocrystals and its doped variants with unique morphologies under optimized reaction conditions for efficient electrochemical and photochromic performances. *Mater. Chem. Phys.* **280**, 125813 (2022). 10.1016/j.matchemphys.2022.125813

[12] Nadkarni, G. S. & Simmons, J. G. Electrical properties of evaporated molybdenum oxide films. *J. Appl. Phys.* **41**, 545–551 (1970). 10.1063/1.1658710

[13] Sabhapathi, V. K. *et al.* Optical absorption studies in molybdenum trioxide thin films. *Phys. Status Solidi (A)* **148**, 167–173 (1995). 10.1002/pssa.2211480114

[14] Ali, H. M., Shokr, E. K., Elkot, S. A. & Mohamed, W. S. Promising molybdenum trioxide films for optically detectable gas sensor and solar cell applications. *Mater. Res. Express* **6**, 126451 (2020). 10.1088/2053-1591/ab6ad0

[15] An, C., Zhang, Y., Guo, H. & Wang, Y. Metal oxide-based supercapacitors: Progress and prospectives. *Nanoscale Adv.* **1**, 4644–4658 (2019). 36133113 10.1039/C9NA00543A PMC9419102

[16] Jafari, A., Anarjan, N. & Jafarizadeh-Malmiri, H. Effects of rotation speed and time, as solvent removal parameters, on the physico-chemical properties of prepared α-tocopherol nanoemulsions using solvent-displacement technique. *Food Sci. Biotechnol.* **29**, 371–378 (2020). 32257520 10.1007/s10068-019-00675-9 PMC7105522

[17] Jadkar, V. *et al.* Synthesis of orthorhombic-molybdenum trioxide (α-MoO_3_) thin films by hot wire-CVD and investigations of its humidity sensing properties. *J. Mater. Sci. Mater. Electron.* **28**, 15790–15796 (2017). 10.1007/s10854-017-7473-6

[18] Han, Y., Rheem, Y., Lee, K.-H., Kim, H. & Myung, N. V. Synthesis and characterization of orthorhombic-MoO_3_ nanofibers with controlled morphology and diameter. *J. Ind. Eng. Chem.* **62**, 231–238 (2018). 10.1016/j.jiec.2017.12.063

[19] Chiang, T. H. & Yeh, H. C. A novel synthesis of α-MoO_3_ nanobelts and the characterization. *J. Alloys Compd.* **585**, 535–541 (2014). 10.1016/j.jallcom.2013.09.137

[20] Song, J., Ni, X., Gao, L. & Zheng, H. Synthesis of metastable h-MoO_3_ by simple chemical precipitation. *Mater. Chem. Phys.* **102**, 245–248 (2007). 10.1016/j.matchemphys.2006.12.011

[21] Chithambararaj, A. & Bose, A. C. Hydrothermal synthesis of hexagonal and orthorhombic MoO_3_ nanoparticles. *J. Alloys Compd.* **509**, 8105–8110 (2011). 10.1016/j.jallcom.2011.05.067

[22] Du, L. *et al.* Synthesis of a novel amphoteric copolymer and its application as a dispersant for coal water slurry preparation. *R. Soc. Open Sci.* **8**, 201480 (2021). 33614083 10.1098/rsos.201480 PMC7890484

[23] Sen, S. K. *et al.* An investigation of 60 Co gamma radiation-induced effects on the properties of nanostructured α-MoO_3_ for the application in optoelectronic and photonic devices. *Opt. Quantum Electron.* **51**, 1–15 (2019). 10.1007/s11082-019-1797-9

[24] Kothaplamoottil Sivan, S. *et al.* Greener assembling of MoO_3_ nanoparticles supported on gum arabic: Cytotoxic effects and catalytic efficacy towards reduction of p-nitrophenol. *Clean Technol. Environ. Policy* **21**, 1549–1561 (2019). 10.1007/s10098-019-01726-9

[25] Varghese, J., Siponkoski, T., Nelo, M., Sebastian, M. T. & Jantunen, H. Microwave dielectric properties of low-temperature sinterable α-MoO_3_. *J. Eur. Ceram. Soc.* **38**, 1541–1547 (2018). 10.1016/j.jeurceramsoc.2017.11.027

[26] Kumar, V. *et al.* Topotactic phase transformation of hexagonal MoO_3_ to layered MoO_3_-II and its two-dimensional (2D) nanosheets. *Chem. Mater.* **26**, 5533–5539 (2014). 10.1021/cm502558t

[27] Ji, F. *et al.* 2D-MoO_3_ nanosheets for superior gas sensors. *Nanoscale* **8**, 8696–8703 (2016). 27053379 10.1039/C6NR00880A

[28] Hu, H., Deng, C., Xu, J., Zhang, K. & Sun, M. Metastable h-MoO_3_ and stable α-MoO_3_ microstructures: Controllable synthesis, growth mechanism and their enhanced photocatalytic activity. *J. Exp. Nanosci.* **10**, 1336–1346 (2015). 10.1080/17458080.2015.1012654

[29] Song, Y., Zhao, Y., Huang, Z. & Zhao, J. Aqueous synthesis of molybdenum trioxide (h-MoO_3_, α-MoO_3_· H_2_O and h-/α-MoO_3_ composites) and their photochromic properties study. *J. Alloys Compd.* **693**, 1290–1296 (2017). 10.1016/j.jallcom.2016.10.092

[30] Ge, H., Kuwahara, Y. & Yamashita, H. Development of defective molybdenum oxides for photocatalysis, thermal catalysis, and photothermal catalysis. *Chem. Commun.* **58**, 8466–8479 (2022). 10.1039/D2CC02658A 35861347

[31] Borgschulte, A. *et al.* Hydrogen reduction of molybdenum oxide at room temperature. *Sci. Rep.* **7**, 40761 (2017). 28094318 10.1038/srep40761 PMC5240095

[32] Bramhaiah, K., Singh, K. K. & John, N. S. Single sea urchin–MoO_3_ nanostructure for surface enhanced Raman spectroscopy of dyes. *Nanoscale Adv.* **1**, 2426–2434 (2019). 36131958 10.1039/C9NA00115H PMC9418698

[33] Bai, H. *et al.* Direct growth of defect-rich MoO_3–x_ ultrathin nanobelts for efficiently catalyzed conversion of isopropyl alcohol to propylene under visible light. *J. Mater. Chem. A* **4**, 1566–1571 (2016). 10.1039/C5TA08603E

[34] Chiang, T. H. & Yeh, H. C. The synthesis of α-MoO_3_ by ethylene glycol. *Materials* **6**, 4609–4625 (2013). 28788350 10.3390/ma6104609 PMC5452848

[35] Bai, S. *et al.* Ultrasonic synthesis of MoO_3_ nanorods and their gas sensing properties. *Sens. Actuators B Chem.* **174**, 51–58 (2012). 10.1016/j.snb.2012.08.015

[36] Salkar, A. V. *et al.* 2D α-MoO_3-x_ truncated microplates and microdisks as electroactive materials for highly efficient asymmetric supercapacitors. *J. Energy Storage* **48**, 103958 (2022). 10.1016/j.est.2022.103958

[37] Chen, S., Xiong, F. & Huang, W. Surface chemistry and catalysis of oxide model catalysts from single crystals to nanocrystals. *Surf. Sci. Rep.* **74**, 100471 (2019). 10.1016/j.surfrep.2019.100471

[38] Patil, M. K., Gaikwad, S. H. & Mukherjee, S. P. Phase-and morphology-controlled synthesis of tunable plasmonic MoO_3–x_ nanomaterials for ultrasensitive surface-enhanced Raman spectroscopy detection. *J. Phys. Chem. C* **124**, 21082–21093 (2020). 10.1021/acs.jpcc.0c06004

[39] Zu, D. *et al.* Oxygen-deficient metal oxides: Synthesis routes and applications in energy and environment. *Nano Res.* **12**, 2150–2163 (2019). 10.1007/s12274-019-2377-9

[40] Jiang, J., Xu, T., Lu, J., Sun, L. & Ni, Z. Defect engineering in 2D materials: Precise manipulation and improved functionalities. *Research* **2019**, 4641739 (2019). 31912036 10.34133/2019/4641739 PMC6944491

[41] Dobrovolsky, A., Merdasa, A., Unger, E. L., Yartsev, A. & Scheblykin, I. G. Defect-induced local variation of crystal phase transition temperature in metal-halide perovskites. *Nat. Commun.* **8**, 34 (2017). 28652597 10.1038/s41467-017-00058-w PMC5484711

[42] Lee, S. A. *et al.* Phase transitions via selective elemental vacancy engineering in complex oxide thin films. *Sci. Rep.* **6**, 23649 (2016). 27033718 10.1038/srep23649 PMC4817049

[43] Akın, Ü. & Şafak, H. Thickness dependence of dispersion parameters of the MoOx thin films prepared using the vacuum evaporation technique. *J. Alloys Compd.* **647**, 146–151 (2015). 10.1016/j.jallcom.2015.06.164

[44] Rouhani, M. *et al.* Photochromism of amorphous molybdenum oxide films with different initial Mo^5+^ relative concentrations. *Appl. Surf. Sci.* **273**, 150–158 (2013). 10.1016/j.apsusc.2013.01.218

[45] Anwar, M. & Hogarth, C. A. Optical properties of amorphous thin films of MoO_3_ deposited by vacuum evaporation. *Phys. Status Solidi (A)* **109**, 469–478 (1988). 10.1002/pssa.2211090213

[46] Xu, M.-F. *et al.* A comprehensive comparison of transition metal oxide MoO_3_ and non-transition metal oxide GeO_2_ in solar cells. *Appl. Phys. A* **124**, 1–5 (2018). 10.1007/s00339-018-2228-7

[47] Zhang, C., Liu, G., Geng, X., Wu, K. & Debliquy, M. Metal oxide semiconductors with highly concentrated oxygen vacancies for gas sensing materials: A review. *Sens. Actuators A Phys.* **309**, 112026 (2020). 10.1016/j.sna.2020.112026

[48] Yuan, H. *et al.* ZnO nanosheets abundant in oxygen vacancies derived from metal-organic frameworks for ppb-level gas sensing. *Adv. Mater.* **31**, 1807161 (2019). 10.1002/adma.201807161 30637791

[49] Fjellvåg, Ø. S., Ruud, A., Sønsteby, H. H., Nilsen, O. & Fjellvåg, H. Crystallization, phase stability, and electrochemical performance of β-MoO_3_ thin films. *Cryst. Growth Des.* **20**, 3861–3866 (2020). 10.1021/acs.cgd.0c00156

[50] Ma, M. *et al.* Oxygen vacancy engineering and superior sensing properties of hematite prepared via a one-step treatment. *Sens. Actuators B Chem.* **339**, 129907 (2021). 10.1016/j.snb.2021.129907

[51] Yang, J. *et al.* Creating oxygen-vacancies in MoO_3-x_ nanobelts toward high volumetric energy-density asymmetric supercapacitors with long lifespan. *Nano Energy* **58**, 455–465 (2019). 10.1016/j.nanoen.2019.01.071

[52] Wang, L., Zhang, G.-H., Sun, Y.-J., Zhou, X.-W. & Chou, K.-C. Preparation of ultrafine β-MoO_3_ from industrial grade MoO_3_ powder by the method of sublimation. *J. Phys. Chem. C* **120**, 19821–19829 (2016). 10.1021/acs.jpcc.6b05982

[53] Lei, Z. *et al.* Recent advances of layered-transition metal oxides for energy-related applications. *Energy Storage Mater.* **36**, 514–550 (2021). 10.1016/j.ensm.2021.01.004

[54] Guo, Y., Xu, K., Wu, C., Zhao, J. & Xie, Y. Surface chemical-modification for engineering the intrinsic physical properties of inorganic two-dimensional nanomaterials. *Chem. Soc. Rev.* **44**, 637–646 (2015). 25406669 10.1039/C4CS00302K

[55] Brachmann, E., Seifert, M., Oswald, S., Menzel, S. B. & Gemming, T. Evaluation of surface cleaning procedures for CTGS substrates for SAW technology with XPS. *Materials* **10**, 1373 (2017). 29189721 10.3390/ma10121373 PMC5744308

[56] de Menezes Atayde, C. & Doi, I. Highly stable hydrophilic surfaces of PDMS thin layer obtained by UV radiation and oxygen plasma treatments. *Phys. Status Solidi C* **7**, 189–192 (2010). 10.1002/pssc.200982419

[57] Lo, M. F., Ng, T. W., Mo, H. W. & Lee, C. S. Direct threat of a UV-ozone treated indium-tin-oxide substrate to the stabilities of common organic semiconductors. *Adv. Funct. Mater.* **23**, 1718–1723 (2013). 10.1002/adfm.201202120

[58] Kumar, N. *et al.* Influence of the substrate temperature on the structural, optical, and electrical properties of tin selenide thin films deposited by thermal evaporation method. *Cryst. Res. Technol.* **45**, 53–58 (2010). 10.1002/crat.200900424

[59] Kumar, N. *et al.* Structure, optical and electrical characterization of tin selenide thin films deposited at room temperature using thermal evaporation method. *J. Nano- Electron. Phys. SumDU* **3**, 117–126 (2011).

