# Supplementary material for: Renewable synthesis of MoO3 nanosheets via low temperature phase transition for supercapacitor application
Source: Sci Rep. 2024 Sep 3;14:20503. doi: 10.1038/s41598-024-69765-x (PMC11372194; doi:10.1038/s41598-024-69765-x)
Supplement: Supplementary file 1 — Supplementary Information. [file 41598_2024_69765_MOESM1_ESM.docx]

**Supplementary Information**

**Renewable Synthesis of MoO_3_ Nanosheets via Low Temperature Phase Transition for Supercapacitor Application**

K. N Amba Sankar ^1 *^, Lokesh Kesavan^2*^, Bikash Saha^3,^ ^4, 8^ M. K Jyolsnaraj ^5^, S. Mohan^6^,

P Nandakumar ^1^, Kallol Mohanta^5, 7 *^, Carita Kvarnström^2 *^

^1^Department of Electronics, PSG College of Arts and Science,

Coimbatore-641014, Tamil Nadu, India.

^2^University of Turku, Department of Chemistry, Materials Chemistry,

Henrikinkatu 2, FI-20014 Turku, Finland

^3^Solid State Physics Division, Bhabha Atomic Research Centre, Mumbai 400085, India,

^4^Homi Bhabha National Institute, Anushakti Nagar, Mumbai 400 094, India.

^5^Nanotech Research Innovation and Incubation Centre (NRIIC),

PSG Institute of Advanced Studies, Avinashi Road, Coimbatore, 641004, Tamil Nadu, India

^6^Physical and Materials Chemistry division,

CSIR- National Chemical Laboratory, Pune- 411008

^7^Senior Research Scientist, Prophecy Sensorlytics LLC,

GN4, Sector V, Salt Lake, Kolkata 700156, West Bengal, India.

^8^Department of Physics and Astronomy, University of Missouri, 223 Physics Building, Columbia, MO 65211, USA.

Corresponding authors’ e-mail: ambasankarji@gmail.com, kmohanta@gmail.com, carkva@utu.fi, lokesa@utu.fi.

| ***h*-MoO3**  **Band frequency (cm^-1^)** | | | | | | **Bonding**  **Structure** |
| --- | --- | --- | --- | --- | --- | --- |
| Our study | Xin Guan  *et al.*^1^ | Bin Hui  *et al.*^2^ | P. Thangasamy  *et al.* ^3^ | Manoj Krishnat Patil *et al.*^4^ | Wenzhi Pan  *et al.*^5^ |  |
| 127 |  | 121 | 121 |  |  | O=Mo=O |
| 150 |  |  | 137 |  |  | O=Mo=O |
| 186 |  | 178 | 177 |  |  | O=Mo=O |
| 241 | 252 | 249 | 253 | 252 |  | Mo–O–Mo |
| 286 |  |  |  |  |  | Mo=O stretching and bending vibration |
| 335 |  | 319 | 319 |  |  | O–Mo–O |
| 376 |  | 398 | 399 |  |  | O–Mo–O |
| 666 | 690 | 691 | 692 | 691 | 690 | O–Mo–O scissoring vibration |
| 820 |  |  |  | 817 |  | Mo_2_–O (mixed phase) |
| 909 | 899 | 902 | 903 | 900 | 901 | Mo =O asymmetrical stretching vibration |
| 993 | 976 | 978 | 977 | 975 | 980 | Mo=O symmetrical stretching |

**Table S1. Comparison of Raman vibration modes (cm^-1^) between reported *h*-MoO_3_ data and our study (*DEST* synthesis)**

| **Raman vibrational modes** | **α-MoO_3_**  **Band frequency (cm^-1^)** | | | | | | **Bonding Structure** |
| --- | --- | --- | --- | --- | --- | --- | --- |
|  | Our study | S.K. Singh Patel *et al*.^6^ | Lupan  *et al.*^7^ | I.C. Silva  *et al.*^8^ | Fangxu Ji  *et al.*^9^ | Xin Guan  *et al.*^1^ |  |
| B_2_g | 117 | 130 | 112 | 116 | - |  | O=Mo=O |
| Ag, B_1_g | 145 | 161 | 154 | 159 | 154 |  | O=Mo=O |
| B_2_g | 195 | 199 | 194 | 197 | - |  | O=Mo=O |
| Ag | - | 219 | 213 | 218 | - |  |  |
| B_3_g | - | 249 | - | 246 | - |  |  |
| B_2_g, B_3_g | 281 | 295 | 280 | 291 | 284 | 289 | O=Mo=O wagging |
| B_1_g, Ag | 335 | 340 | 333 | 338 | 336 | 340 | O–Mo–O bending |
| Ag | 376 | 381 | 375 | 379 | 378 | 380 | O–Mo–O scissoring |
| Ag, B_1_g | - | 482 | 469 | 471 | - |  |  |
| B_2_g, B_3_g | 666 | 666 | 662 | 666 | 671 | 666 | O–Mo–O asymmetric stretching |
| Ag, B_1_g | 820 | 820 | 815 | 819 | 821 | 822 | Mo–O–Mo symmetric stretching |
| Ag, B_1_g | 993 | 995 | 991 | 995 | 999 | 994 | Mo–O–Mo asymmetric stretch of the terminal oxygen atoms |

**Table S2. Comparison of Raman vibration modes (cm^-1^) between reported α-MoO_3_ data and our study (*DEST* synthesis)**


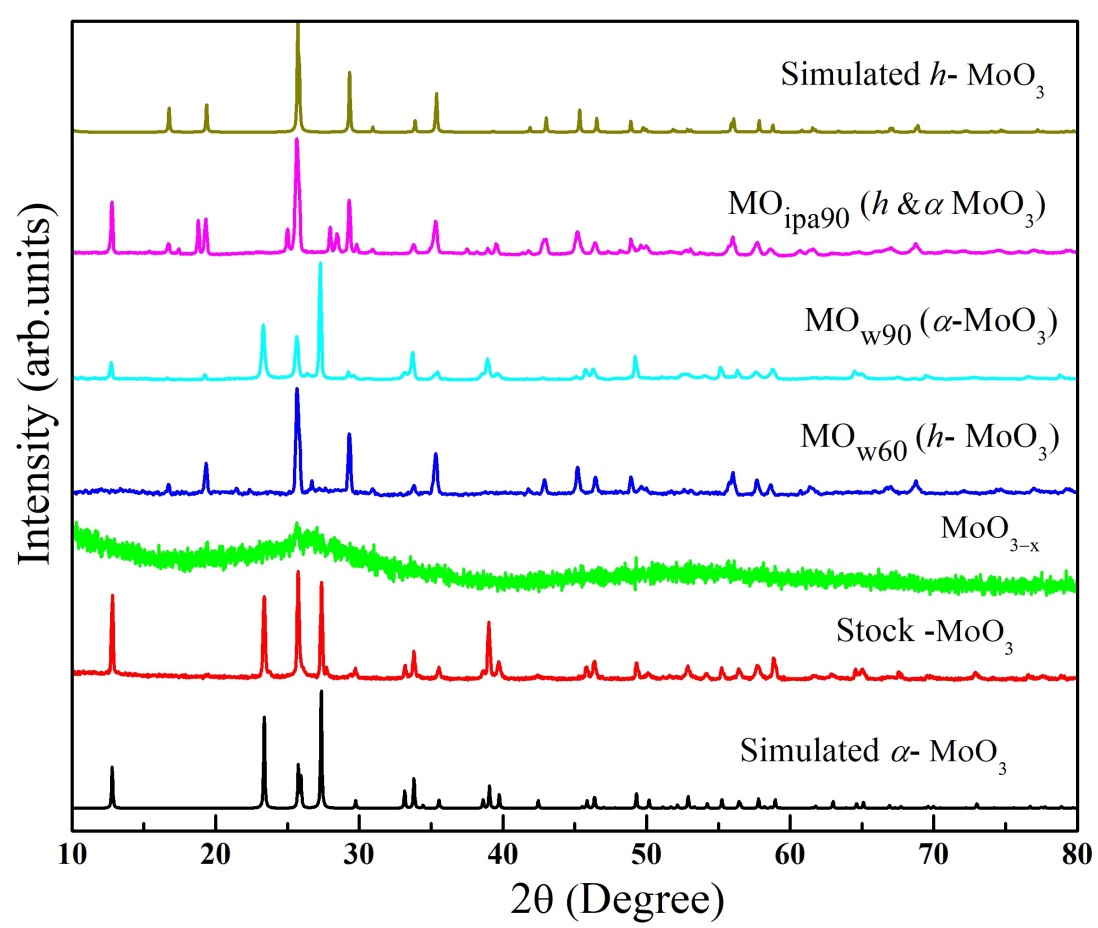


**Figure. S1. XRD showing the amorphous MoO_3-x_ (obtained after PVD step) in comparison with other crystalline MoO_3_ polymorphs (obtained after USE-VST steps) .**

| **Samples** | ***h*-MoO_3_ (MO_w60_)** | | | ***α*-MoO_3_ (MO_w90_)** | | |
| --- | --- | --- | --- | --- | --- | --- |
| **Atoms** | ***x*/*a*** | ***y*/*b*** | ***z*/*c*** | ***x*/*a*** | ***y*/*b*** | ***z*/*c*** |
| Mo | 0.891(4) | 0.354(5) | 0.75 | 0.058(2) | 0.102(4) | 0.25 |
| O1 | 0.921(3) | 0.421(3) | 0.25 | 0.490(9) | 0.433(4) | 0.25 |
| O2 | 0.717(3) | 0.223(3) | 0.75 | 0.566(8) | 0.098(3) | 0.25 |
| O3 | 0.992(4) | 0.261(6) | 0.75 | 0.096(5) | 0.215(2) | 0.25 |

**Table S3. The Rietveld refined crystal structural parameters (fractional atomic coordinates) of *DEST*-made *h*-MoO_3_ (MO_w60_) & *α*-MoO_3_ (MO_w90_) samples.**

| **Sample** | **Mixed Phases (MO_ipa90_)** | | | | | |
| --- | --- | --- | --- | --- | --- | --- |
|  | ***h*-MoO_3_** | | | ***α*-MoO_3_** | | |
| **Atoms** | ***x*/*a*** | ***y*/*b*** | ***z*/*c*** | ***x*/*a*** | ***y*/*b*** | ***z*/*c*** |
| Mo | 0.889(2) | 0.354(1) | 0.75 | 0.191(4) | 0.094(3) | 0.25 |
| O1 | 0.915(2) | 0.424(2) | 0.25 | 0.697(5) | 0.383(4) | 0.25 |
| O2 | 0.979(3) | 0.25606(3) | 0.75 | 0.828(3) | 0.091(5) | 0.25 |
| O3 | 0.851(3) | 0.010(3) | 0.75 | 0.929(5) | 0.521(3) | 0.25 |

**Table S4. The Rietveld refined crystal structural parameters (fractional atomic coordinates) of *h*-MoO_3_ and *α*-MoO_3_ in the mixed phase, in *DEST*-made MO_ipa90_ sample.**


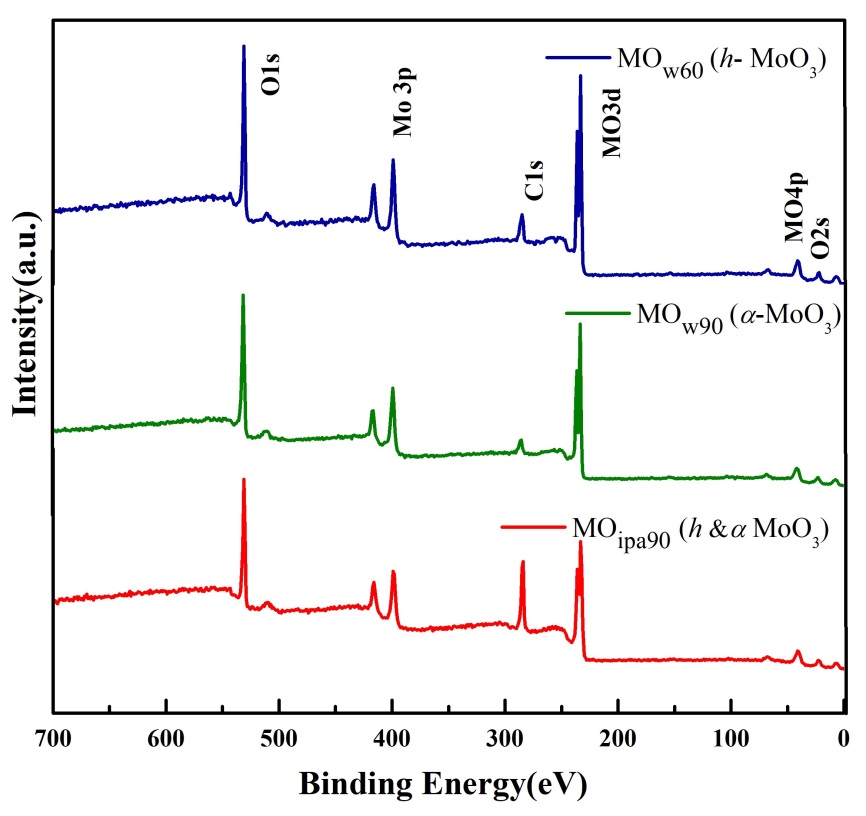


**Figure. S2. XPS survey spectra of *DEST*-made MoO_3_ polymorphs.**

| **S.No.** | **Sample ID** | **Mo-3d**  **Peak Position** | | **O-1s**  **Peak Position** | **O-1s**  **Peak Area**  **( × 10^4^)** |
| --- | --- | --- | --- | --- | --- |
|  |  | **3d_3/2_** | **3d_5/2_** |  |  |
| 1. | MO_w60_ (*h*-MoO_3_) | 236.53 | 233.38 | 531.28 | 20.93 |
| 2. | MO_w90_ (α-MoO_3_) | 236.98 | 233.78 | 531.78 | 42.76 |
| 3. | MO_ipa90_ (Mixed phases of 72.6% *h*-MoO_3_ & 27.4% *α*-MoO_3_) | 235.98 | 232.88 | 530.78 | 24.92 |

**Table. S5. Mo-, O- peak positions & area under the O-1s peak in XPS for *DEST*-made MoO_3_ polymorphs.**


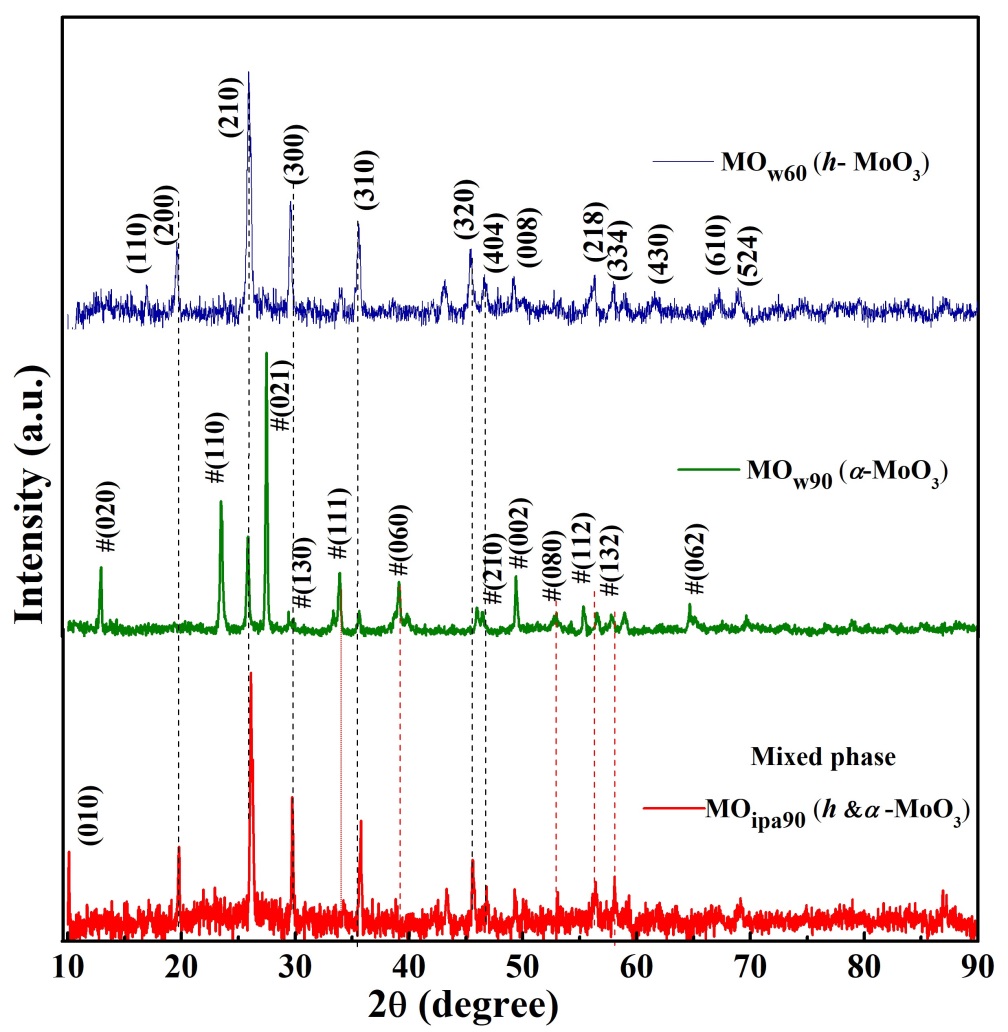


**Figure. S3. XRD showing the *DEST*-made 2D MoO_3_ materials from different batch of synthesis - Reproduced.**

| **S.No** | **XRD (Xpert highscore Reference No)** | | | | | |
| --- | --- | --- | --- | --- | --- | --- |
|  | **021-0569** | | **001-0706** | | **021-0561** | |
|  | **MO_W60_**  **(*h*-MoO_3_)** | | **MO_W90_**  **(*α*-MoO_3_)** | | **MO_ipa90_**  **(*h*-MoO_3_)** | |
|  | **Diffraction angle (****2θ)** | | | | | |
|  | **Batch 1** | **Batch 2** | **Batch 1** | **Batch 2** | **Batch 1** | **Batch 2** |
| 1 | 9.6 | - | 12.8 | 12.8 | 9.6 | 10.1 |
| 2 | 16.7 | - | 23.3 | 23.4 | 12.7 | - |
| 3 | 19.3 | 19.6 | 25.7 | 25.8 | 16.7 | - |
| 4 | 25.6 | 25.9 | 27.3 | 27.4 | 19.3 | 19.7 |
| 5 | 29.2 | 29.5 | 29.3 | 29.7 | 25 | - |
| 6 | 30.8 | - | 33.7 | 33.8 | 25.6 | 26.1 |
| 7 | 33.8 | 33.8 | 38.9 | 39 | 29.3 | 29.7 |
| 8 | 35.3 | 35.5 | 45.8 | 45.9 | 35.3 | 35.7 |
| 9 | 42.9 | - | 46.4 | - |  |  |
| 10 | 45.1 | 45.3 | 49.2 | 49.3 |  |  |
| 11 | 46.3 | 46.5 | 52.7 | 52.9 |  |  |
| 12 | 48.9 | 49.2 | 55.2 | 55.2 |  |  |
| 13 | 56 | 56.3 | 56.3 | 56.5 |  |  |
| 14 | 57.6 | 57.9 | 57.6 | 57.7 |  |  |
| 15 | 58.6 | - | 58.9 | 58.9 |  |  |
| 16 | 61.4 | 61.5 | 64.5 | 64.6 |  |  |
| 17 | 67 | 67.2 | 69.6 | 69.6 |  |  |
| 18 | 68.7 | 68.8 |  |  |  |  |

**Table. S6. Comparison of X- Ray Diffraction angle (2θ) between two batches of samples (*DEST* syntheses). Batch 1- Fig. 4, Batch 2-Fig.S3.**

| **References** | **Preparation method** | **Transition temperature (°C)** | **Change of Phase** |
| --- | --- | --- | --- |
| Hans-Joachim et al.^10^ | Acidification | 425 °C | h → α |
| Ramana et al.^11^ | Precipitation | 400 °C | h → α |
| Song et al.^12^ | Precipitation | 376 °C | h → α |
| Wu et al.^13^ | Sonochemical | 418 °C | h → α |
| Chithambaraj et al.^14^ | Hydrothermal | 430 °C | h → α |
| S.R. Dhage et al.^15^ | Probe sonication | 400 °C | h → α |
| V M Jain et al^16^ | Chemical method | 400 °C | h → α |
| Shouli Bai^17^ | Ultrasonic approach | 436 °C | h → α |
| Our present study | *DEST*  (Water as solvent) | 90 °C | h → α |

**Table S7. Comparative literature on the phase transition temperatures of MoO_3_ (*h* → *α* - MoO_3_).**

| **Refer-ences** | **Seed materials** | **Method** | | **Temp** | **Crystalline phase** | **Lattice parameters** | | | **Band gap**  **(eV)** |
| --- | --- | --- | --- | --- | --- | --- | --- | --- | --- |
|  |  |  |  |  |  | ***a*** | ***b*** | ***c*** |  |
| Our work | Commercial MoO_3_ Powder | *D*  *E*  *S*  *T* | Water | 60 °C | *h*-MoO_3_ | 10.61 | 10.61 | 3.72 | 2.94 |
|  |  |  | Water | 90 °C | *α*-MoO_3_ | 3.95 | 3.95 | 3.72 | 2.45 |
|  |  |  | *iso*-propanol | 90 °C | Mixed phase (*h/α*)-MoO_3_ | 10.61  /3. 98 | 10.61  /13.86 | 3.72 /3.71 | 1.5 |
| ^14^ | Ammonium molybdate | Hydrothermal synthesis | | 90 °C | *h*-MoO_3_ | 10.47 | 10.47 | 14.91 | 3.01 |
|  |  |  |  | 150 °C | Mixed phase (*h/α*)-MoO_3_ | 10.54  /3.78 | 10.54 /13.80 | 14.90  /3.25 | 3.24 |
|  |  |  |  | 210 °C | *α*-MoO_3_ | 3.78 | 13.77 | 3.25 | 3.15 |
| ^17^ | Ammonium hepta-molybdate tetrahydrate | Probe ultrasonic route | | 300 °C | *h*-MoO_3_ | 10.53 | 10.53 | 14.87 | - |
| ^12^ | Sodium molybdate | Solution  Process  (MoO_4_ ^2-^: HCl) | | (1:8) ratio  90 °C | *h*-MoO_3_ | - | - | - | 2.86 |
|  |  |  |  | (1:11) ratio  90 °C | Mixed phase (*h/α*)-MoO_3_ | - | - | - | 1.55 |
|  |  |  |  | (1:10) ratio  90 °C | *α*-MoO_3_.H_2_O | - | - | - | 3.26 |
| ^18^ | Ammonium hepta-molybdate | Hydrothermal synthesis  (NH_4_)_6_Mo_7_O_2_  ·4H_2_O: HNO_3_)  in a ratio | | (2:1) ratio  149.85 °C | *h*-MoO_3_ | - | - | - | 2.81 |
|  |  |  |  | (8:1) ratio  149.85 °C | Mixed phase (*h/α*)-MoO_3_ | - | - | - | 2.75 |
| ^11^ | Ammonium paramolybdate | Chemical  precipitation | | 300 °C | *h*-MoO_3_ | - | - | - | 3.01 |
| ^16^ | Ammonium hepta-molybdate | Solution  Process | | 400 °C | *α*-MoO_3_ | - | - | - | 2.95 |
| ^19^ | Commercial MoO_3_ Powder | Spray pyrolysis | | 300 °C | *α*-MoO_3_ | - | - | - | 2.85 |
|  |  |  |  | 350 °C | *α*-MoO_3_ | - | - | - | 2.81 |
|  |  |  |  | 400 °C | *α*-MoO_3_ | - | - | - | 2.56 |
|  |  |  |  | 450 °C | *α*-MoO_3_ | - | - | - | 2.72 |

**Table. S8. Discrepancies between the reported methods and our method (*DEST*) in terms of phase transition temperatures and crystal structures of MoO_3_ polymorphs.**

| **S.**  **No.** | **References** | **Material** | **Electrolyte** | **No. of Electrodes** | **Scan rate** | **Specific capacitance F/g** | **Synthesis Temperature** |
| --- | --- | --- | --- | --- | --- | --- | --- |
| 1. | Guru Prakash et al.^20^ | *α*-MoO_3_ | 1M  Na_2_SO_4_ | 3 | 1 mA/g | 176 | 500 ℃ |
| 2. | Juan C. Icaza et al.^21^ | *α*-MoO_3_ | 0.5 M BeSO_4_ | 3 | 25 A/g | 169 | Commercial sample |
| 3. | M. Y. Ho et al.^22^ | MoO_3_/  graphene | 1M Na_2_SO_3_ | 2 | 5 mV/s | 148 | 500 °C |
| 4. | S. A. Khalate et al.^19^ | *α*-MoO_3_ | 0.5 M  Na_2_SO_4_ | 3 | 100 mV/s | 118.79 | 400 °C |
| 5 | Our present study | *α*-MoO_3_ | 1M  Na_2_SO_4_ | 3 | 20 mV/s | 256 | 90 °C |

**Table. S9. Comparison of capacitive *α*-MoO_3_ coated** **electrodes’ performance in recent reports.**

| **S.**  **No.** | **Composite** | **Electrolyte** | **Specific capacitance F/g** | **References** |
| --- | --- | --- | --- | --- |
| 1. | MoS_2_/rGO | 1 M HClO4 | 148 F g^−1^ | ^23^ |
| 2. | V2CT_x_ MXene | Seawater | 181.1 F g^−1^ | ^24^ |
| 3. | NiSe_2_ | 1 M KOH | 75 F g^−1^ | ^25^ |
| 4. | MoS_2_ | 1 M Na2SO4 | 138 F g^−1^ | ^26^ |
| 5. | N-doped graphene-CNT | 6 M KOH | 180 F g^−1^ | ^27^ |
| 6. | Few layer graphene | 1 M Na2SO4 | 180 F g^−1^ | ^28^ |

**Table. S10. Comparison of capacitive non- MoO_3_ 2D electrode materials performance.**

| ***DEST* Synthesis of MoO_3_ polymorphs 2D nanosheets** | |
| --- | --- |
| 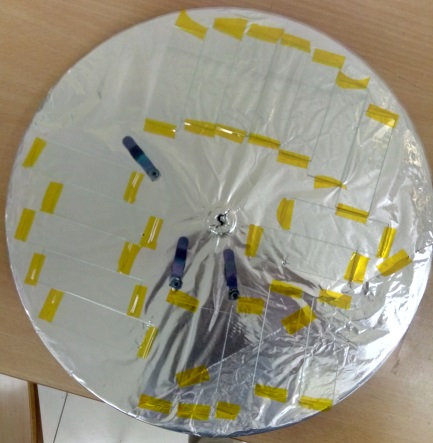 | 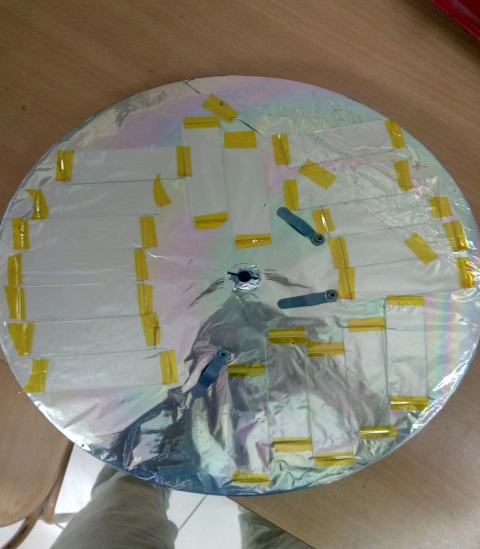 |
| Clean glass substrates mounted on dial plate | Physical vapor of MoO_3_ deposited  (PVD) glass substrates |
|  |  |
| 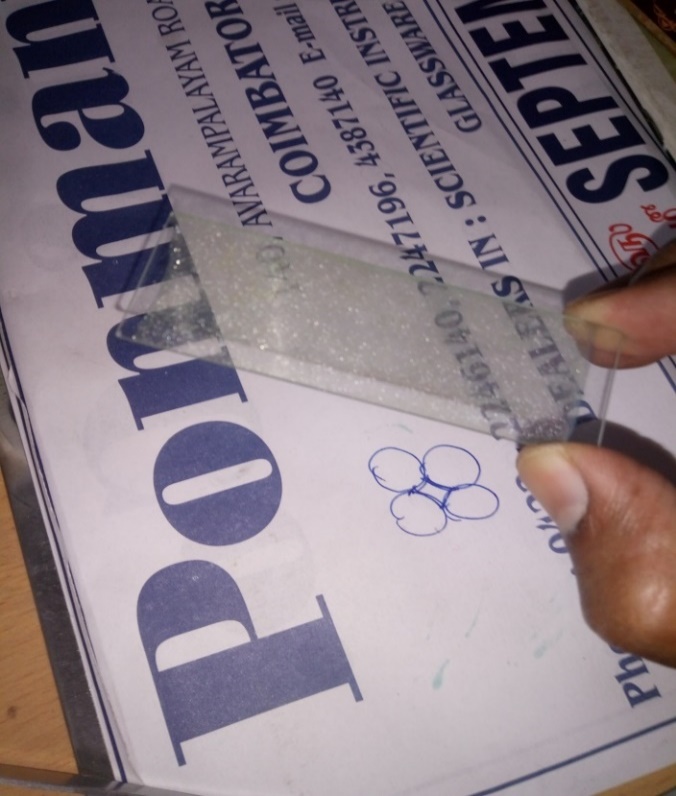 | 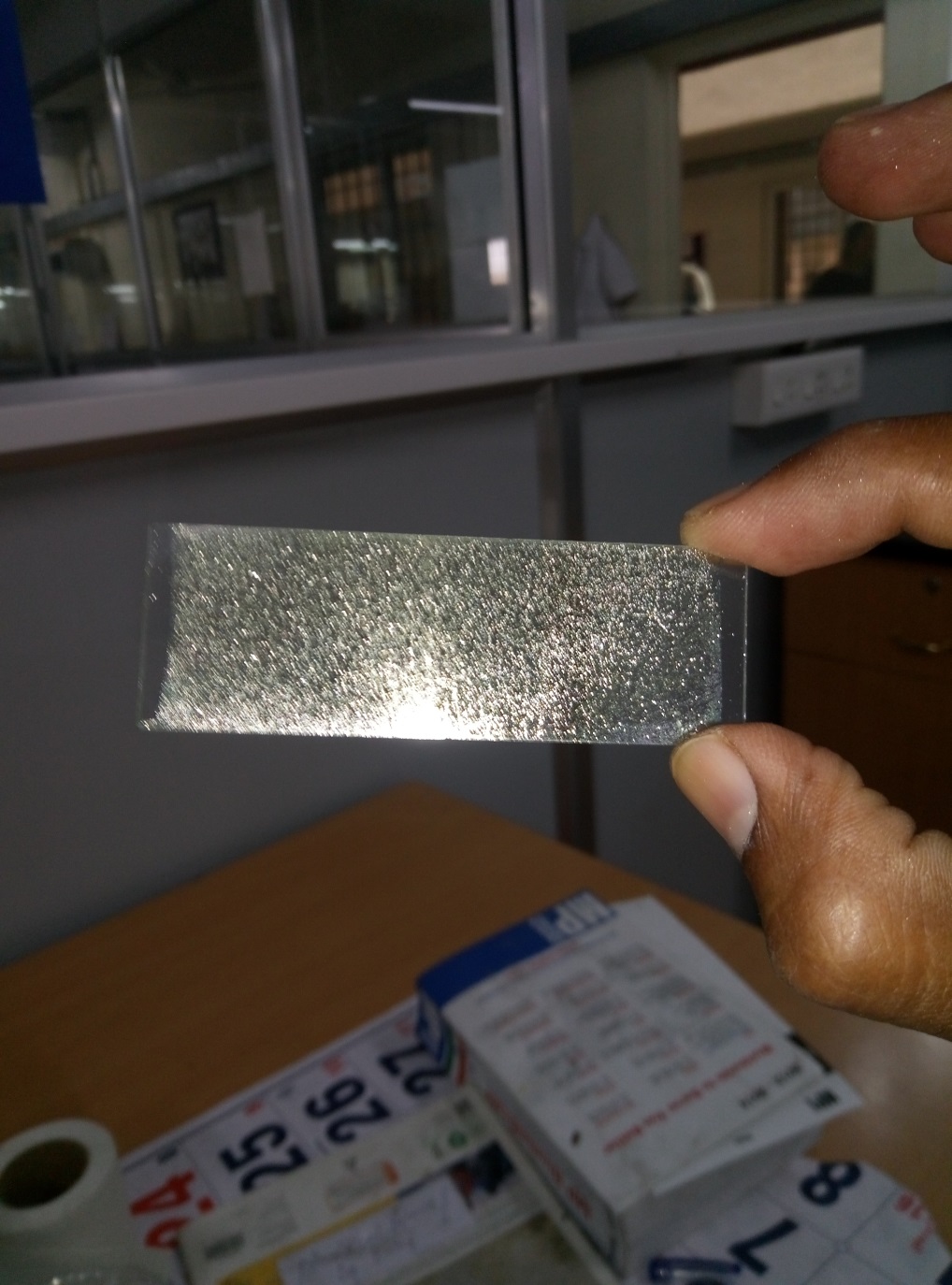 |
| MoO_3-x_ thin film coated glass substrates | |
| 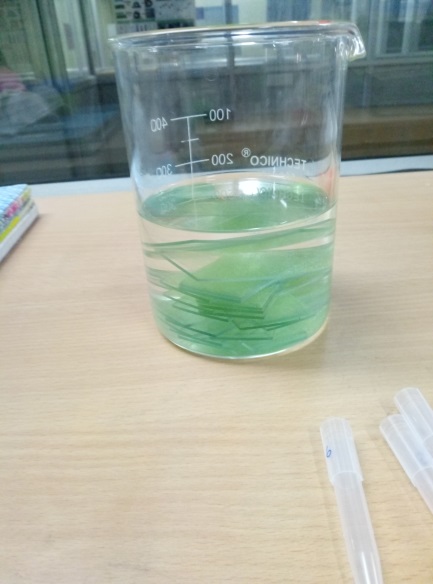 | 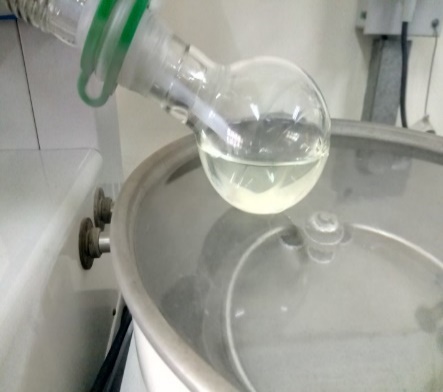 |
| Substrates immersed in protic polar solvent for ultrasonic extraction (USE) of MoO_3-x_ Nanopowder | Recrystallization of MoO_3-x_ by vacuum assisted solvothermal treatment (VST) |
| 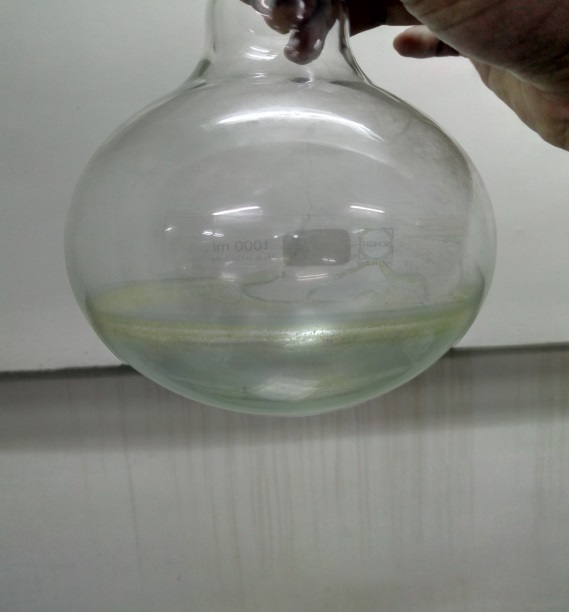 | 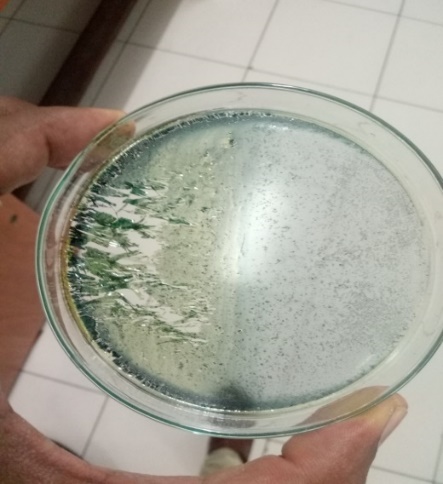 |
| Crystalline MoO_3_ concentrate | 2D MoO_3_ nanosheets (Dried) |

**Figure. S4. Flow diagram of the *DEST* Synthesis of** **MoO_3_ polymorph 2D nanosheets.**

**References**

1 Guan, X. *et al.* Charge separation and strong adsorption-enhanced MoO 3 visible light photocatalytic performance. *Journal of Materials Science* **55**, 5808-5822 (2020).

2 Hui, B. *et al.* h-MoO 3 microrods grown on wood substrates through a low-temperature hydrothermal route and their optical properties. *Journal of Materials Science: Materials in Electronics* **28**, 3264-3271 (2017).

3 Thangasamy, P., Shanmugapriya, V. & Sathish, M. One-dimensional growth of hexagonal rods of metastable h-MoO3 using one-pot, rapid and environmentally benign supercritical fluid processing. *Physica E: Low-dimensional Systems and Nanostructures* **99**, 189-193 (2018).

4 Patil, M. K., Gaikwad, S. H. & Mukherjee, S. P. Phase-and morphology-controlled synthesis of tunable plasmonic MoO3–x nanomaterials for ultrasensitive surface-enhanced raman spectroscopy detection. *The Journal of Physical Chemistry C* **124**, 21082-21093 (2020).

5 Pan, W. *et al.* Structure, optical, and catalytic properties of novel hexagonal metastable h-MoO3 nano-and microrods synthesized with modified liquid-phase processes. *Chemistry of Materials* **22**, 6202-6208 (2010).

6 Patel, S. K. S. *et al.* Synthesis of α-MoO3 nanofibers for enhanced field-emission properties. *Adv. Mater. Lett.* **9**, 585-589 (2018).

7 Lupan, O. *et al.* Investigation of optical properties and electronic transitions in bulk and nano-microribbons of molybdenum trioxide. *Journal of Physics D: Applied Physics* **47**, 085302 (2014).

8 de Castro Silva, I., Reinaldo, A. C., Sigoli, F. A. & Mazali, I. O. Raman spectroscopy-in situ characterization of reversibly intercalated oxygen vacancies in α-MoO 3. *RSC advances* **10**, 18512-18518 (2020).

9 Ji, F. *et al.* 2D-MoO 3 nanosheets for superior gas sensors. *Nanoscale* **8**, 8696-8703 (2016).

10 Lunk, H.-J. *et al.* “Hexagonal Molybdenum Trioxide”— Known for 100 Years and Still a Fount of New Discoveries. *Inorg. Chem.* **49**, 9400-9408 (2010).

11 Ramana, C. V. *et al.* Low-temperature synthesis of morphology controlled metastable hexagonal molybdenum trioxide (MoO3). *Solid state communications* **149**, 6-9 (2009).

12 Song, Y., Zhao, Y., Huang, Z. & Zhao, J. Aqueous synthesis of molybdenum trioxide (h-MoO3, α-MoO3· H2O and h-/α-MoO3 composites) and their photochromic properties study. *Journal of Alloys and Compounds* **693**, 1290-1296 (2017).

13 Wu, Z., Wang, D., Liang, X. & Sun, A. Ultrasonic-assisted preparation of metastable hexagonal MoO3 nanorods and their transformation to microbelts. *Ultrason. Sonochem.* **18**, 288-292 (2011).

14 Chithambararaj, A. & Bose, A. C. Hydrothermal synthesis of hexagonal and orthorhombic MoO3 nanoparticles. *Journal of Alloys and Compounds* **509**, 8105-8110 (2011).

15 Dhage, S. R., Hassan, M. S. & Yang, O. B. Low temperature fabrication of hexagon shaped h-MoO3 nanorods and its phase transformation. *Mater. Chem. Phys.* **114**, 511-514 (2009).

16 Jain, V. M., Shah, D. V., Patel, K. K. & Doshi, Y. 1 edn 012052 (IOP Publishing).

17 Bai, S. *et al.* Ultrasonic synthesis of MoO3 nanorods and their gas sensing properties. *Sensors and Actuators B: Chemical* **174**, 51-58 (2012).

18 Lu, W., Li, H.-x. & Xue, Z.-l. Synthesis of h-MoO3 nanorods and h-/α-MoO3 composites and their photocatalytic performance. *Transactions of Nonferrous Metals Society of China* **33**, 2155-2167 (2023).

19 Khalate, S. A., Kate, R. S., Pathan, H. M. & Deokate, R. J. Structural and electrochemical properties of spray deposited molybdenum trioxide (α-MoO3) thin films. *Journal of Solid State Electrochemistry* **21**, 2737-2746 (2017).

20 Prakash, N. G. *et al.* High performance one dimensional α-MoO3 nanorods for supercapacitor applications. *Ceram. Int.* **44**, 9967-9975 (2018).

21 Icaza, J. C. & Guduru, R. K. Characterization of α-MoO3 anode with aqueous beryllium sulfate for supercapacitors. *J. Alloys Compd.* **726**, 453-459 (2017).

22 Ho, M. Y., Khiew, P. S., Isa, D., Chiu, W. S. & Chia, C. H. Solvothermal synthesis of molybdenum oxide on liquid-phase exfoliated graphene composite electrodes for aqueous supercapacitor application. *Journal of Materials Science: Materials in Electronics* **28**, 6907-6918 (2017).

23 da Silveira Firmiano, E. G. *et al.* Supercapacitor electrodes obtained by directly bonding 2D MoS2 on reduced graphene oxide. *Advanced Energy Materials* **4**, 1301380 (2014).

24 He, H. *et al.* Two-dimensional vanadium carbide (V2CTx) MXene as supercapacitor electrode in seawater electrolyte. *Chinese Chemical Letters* **31**, 984-987 (2020).

25 Arul, N. S. & Han, J. I. Facile hydrothermal synthesis of hexapod-like two dimensional dichalcogenide NiSe2 for supercapacitor. *Materials Letters* **181**, 345-349 (2016).

26 Sanger, A., Malik, V. K. & Chandra, R. One step sputtered grown MoS2 nanoworms binder free electrodes for high performance supercapacitor application. *International Journal of Hydrogen Energy* **43**, 11141-11149 (2018).

27 You, B., Wang, L., Yao, L. & Yang, J. Three dimensional N-doped graphene–CNT networks for supercapacitor. *Chem. Commun.* **49**, 5016-5018 (2013).

28 Li, Z. J., Yang, B. C., Zhang, S. R. & Zhao, C. M. Graphene oxide with improved electrical conductivity for supercapacitor electrodes. *Appl. Surf. Sci.* **258**, 3726-3731 (2012).
